# Evolution of the metabolome in response to selection for increased immunity in populations of *Drosophila melanogaster*

**DOI:** 10.1371/journal.pone.0188089

**Published:** 2017-11-17

**Authors:** Navdeep Gogna, Rakesh Sharma, Vanika Gupta, Kavita Dorai, N. G. Prasad

**Affiliations:** 1 Department of Physical Sciences, Indian Institute of Science Education & Research IISER, Mohali, Punjab, India; 2 Department of Biological Sciences, Indian Institute of Science Education & Research IISER, Mohali, Punjab, India; Inha University, REPUBLIC OF KOREA

## Abstract

We used NMR-based metabolomics to test two hypotheses–(i) there will be evolved differences in the metabolome of selected and control populations even under un-infected conditions and (ii) post infection, the metabolomes of the selected and control populations will respond differently. We selected replicate populations of *Drosophila melanogaster* for increased survivorship (I) against a gram-negative pathogen. We subjected the selected (I) and their control populations (S) to three different treatments: (1) infected with heat-killed bacteria (*i*), (2) sham infected (*s*), and (3) untreated (*u*). We performed 1D and 2D NMR experiments to identify the metabolic differences. Multivariate analysis of the metabolic profiles of the untreated (I*u* and S*u*) flies yielded higher concentrations of lipids, organic acids, sugars, amino acids, NAD and AMP in the I*u* treatment as compared to the S*u* treatment, showing that even in the absence of infection, the metabolome of the I and S regimes was different. In the S and I regimes, post infection/injury, concentration of metabolites directly or indirectly associated with energy related pathways (lipids, organic acids, sugars) declined while the concentration of metabolites that are probably associated with immune response (amino acids) increased. However, in most cases, the I regime flies had a higher concentration of such metabolites even under un-infected conditions. The change in the metabolite concentration upon infection/injury was not always comparable between I and S regimes (in case of lactate, alanine, leucine, lysine, threonine) indicating that the I and S regimes had evolved to respond differentially to infection and to injury.

## Introduction

Given the ubiquitous nature of pathogens, the ability to mount an effective immune response is an important determinant of the fitness of an organism. However, the maintenance and deployment of an immune response is energetically costly and therefore, it is not surprising that in most animals, immune response and metabolic regulation are tightly coupled [1–2]. Infection, in most animals including flies and humans, leads to the disruption of metabolism. The shifting of resources from metabolism towards the energy intensive immune response is claimed to be central to these changes. Several lines of evidence are in line with this claim. Prolonged infection and immune activation can lead to metabolic dysregulation causing wasting in animals [3]. Infection with certain bacteria and fungi leads to loss of metabolic energy reserves and decline in triglyceride levels in flies [4–6]. In *Drosophila* larvae, systemic infection leads to increased transcription and depletion of nutrient stores, thereby affecting the growth of the organism [4]. In many cases, the molecular mechanism of the switch regulating investment in immunity versus metabolism is also very well worked out. For example, MEF 2, a transcription factor acts as a critical switch between energy storage and immune function in adult *Drosophila* fat bodies [2] while Foxo dependent transcription mediate immunity versus growth in larvae [5]. Using microarrays, Felix et al [7] found that the variation in bacterial clearance in inbred lines of *Drosophila* was not majorly explained by variation in immunity related gene expression. Instead, a large amount of the variation in bacterial clearing ability was associated with genes related to energy metabolism including the insulin/TOR signaling pathway. Taken together, these data clearly indicate a genetic basis for the strong coupling of immune response and metabolism.

Given that (a) immune response is an important component of fitness, (b) immune response involves alteration in metabolism and (c) variation in metabolism related genes, to a large extent, explains variation in immune response, it is reasonable to expect that under directional selection, both immune response and the associated metabolic pathways will evolve. While many studies have addressed the relationship between immune response and the metabolome (for example [6]), none have tracked the simultaneous evolution of immune response and the metabolome. In the present study, we address this issue by performing high-resolution nuclear magnetic resonance (NMR)-based metabolomics on replicate populations of *Drosophila melanogaster* selected for increased survival in the face of a bacterial infection. When a population consistently faces infection across generations, then, it is quite possible that the basal metabolome (ie metabolome in the un-infected state) evolves to be different from the controls in such a way that the selected population is better prepared to handle infection. Additionally/alternatively, the selected population can evolve with respect to the way in which its metabolome changes post infection. Therefore, in the present study we specifically asked two questions-

1. Is the metabolome of the selected populations different from that of the control populations even under un-infected conditions?
2. Are the changes in the metabolome of the selected populations post infection similar to the changes in the metabolome of the control populations post infection?

Metabolomics describes correlations between the metabolome of an organism and its phenotypic state. High-resolution NMR-based metabolomics combined with pattern recognition methods and multivariate statistical analysis, has provided interesting insights into the *Drosophila* metabolome. NMR-based entometabolomics studies have helped reveal hitherto hidden aspects of insect behavior and development [8–12], immunity and infection [6], response to temperature stress [13–17], temperature acclimation [18], bacteria-insect symbiosis [19], effects of inbreeding [20], as well as circadian rhythms [21,22]. Such NMR-based studies have yielded clear metabolomic signatures of adaptation to temperature [23], hypoxia [24–25], and selection for increased lifespan [26].

In the present study we used 1D and 2D high-resolution NMR experiments to profile the metabolites that contribute significantly to the differences between the immune selected (I) and the control (S) fly populations, under three different treatments namely: infection by a pathogen (*i*), injury by pricking with a needle containing a buffer(*s*) and unhandled control treatment (*u*). Thus we had six different combinations of treated flies (I*i*, I*s* and I*u* for I population and S*i*, S*s* and S*u* for S population). We were able to identify a large number of metabolites in the spectra of all the six different combinations of treatments. Of these, we found 12 metabolites that had evolved to have different concentrations in the I and S regimes at the basal level (i.e I*u* and S*u* populations). Upon prick injury and infection by a pathogen, both I and S regimes responded differently. For some metabolites, both I and S regimes showed similar changes in the metabolome whereas for others, the change in the metabolite concentration was different for both I and S regimes. Our results highlight the important role that metabolism plays in an organism’s response to infection by a pathogen and to injury. Our results are the first to show the evolution of the metabolome in response to selection for increased immunity against a bacterial pathogen.

## Results

### Metabolite fingerprinting

Both 1D and 2D NMR spectra were recorded to identify the presence of a wide variety of metabolites in *Drosophila melanogaster*. The metabolites were assigned specific resonances based on their chemical shift values matching with the values of pure compounds recorded in spectral databases such as BMRB and MMCD. The list of metabolites identified using their chemical shift values and scalar coupling patterns (S1 Table and S1 Fig) were also confirmed by homonuclear 2D experiments such as COSY and TOCSY and heteronuclear 2D experiments such as HSQC and HMQC (S2 and S3 Figs).

### Statistical analysis

**Fig 1:**
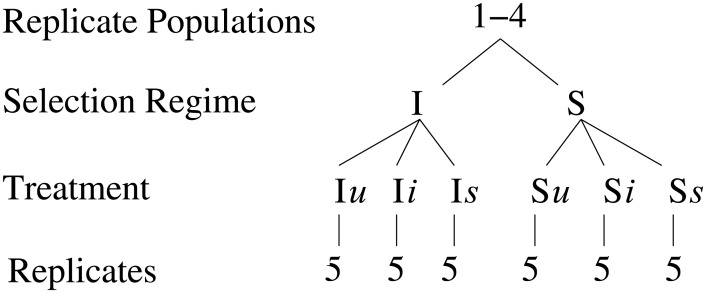
Experimental design for the NMR metabolomics experiments. Four independent blocks, each having two regimes I and S were subjected to three treatments. Each treatment had 5 replicates.

**Fig 2:**
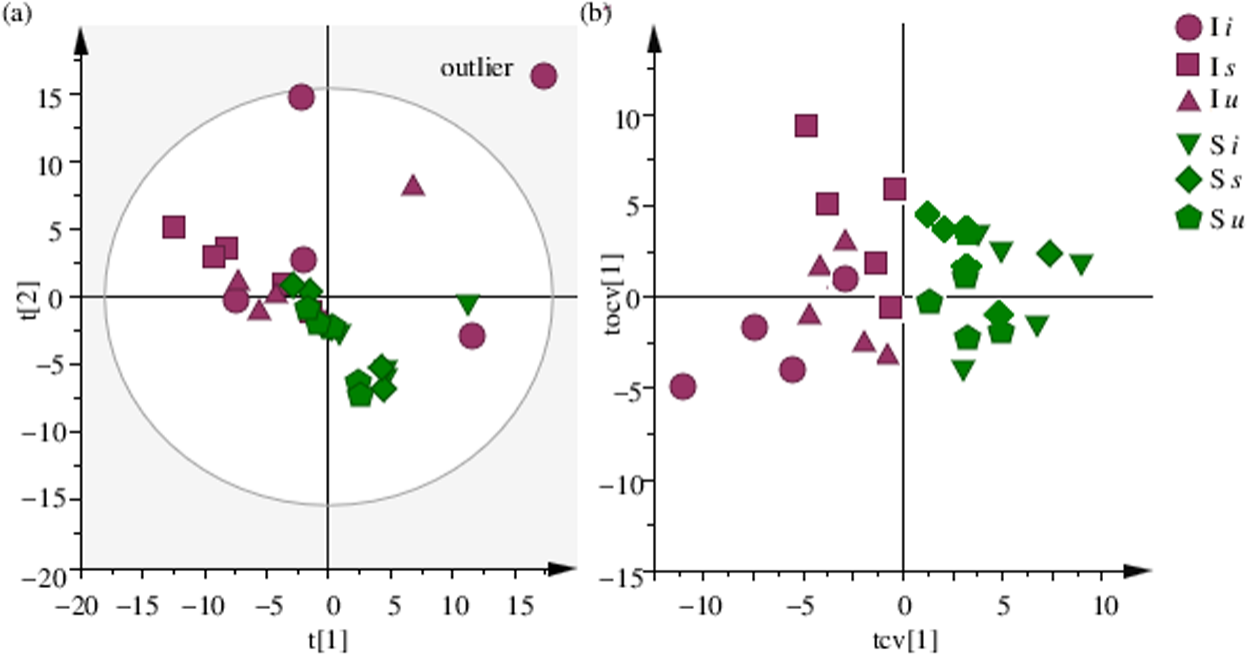
PCA and OPLS-DA score plot. **(a)** Principal component analysis (PCA) score plot of 1H NMR spectra of all six treatments, with component 1 showing 38.9% and component 2 showing 25.4% of the variation and (b) the corresponding cross-validated OPLS-DA score plot after removal of the outlier.

**Fig 3:**
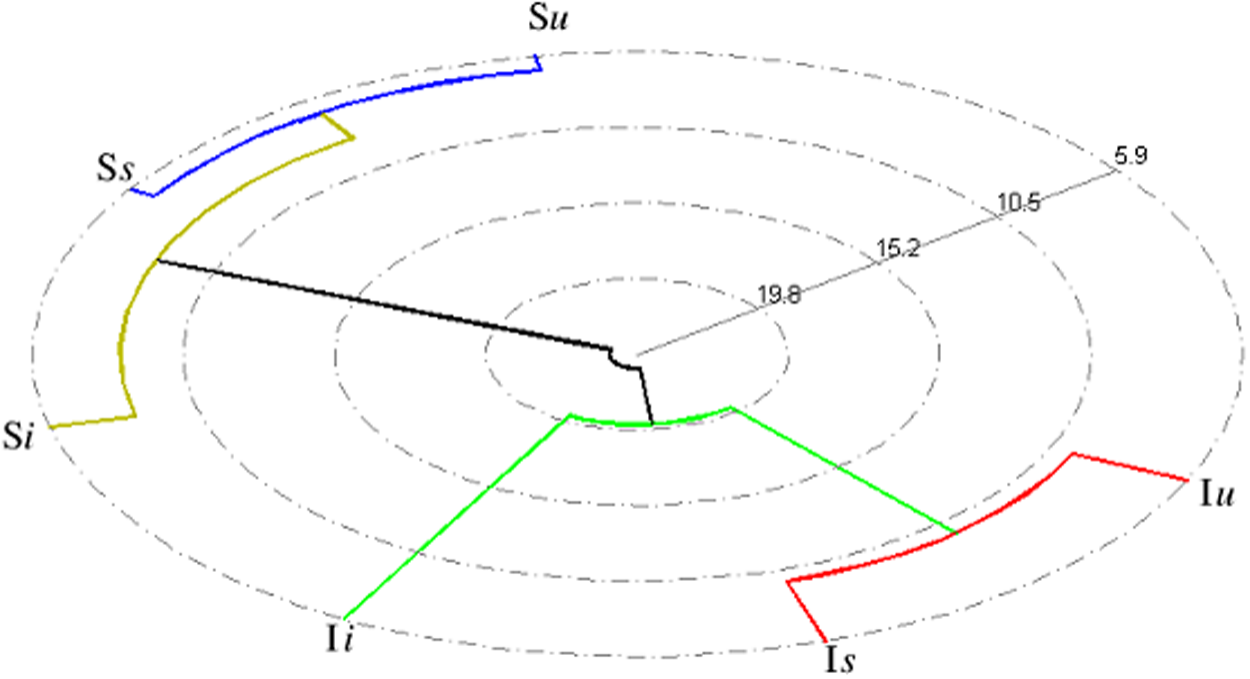
Polar dendrogram. Polar dendrogram showing differences between all the I treatments (I*u*, I*i* and I*s*) and all the S treatments (S*u*, S*i* and S*s*). Each treatment is an average of five replicates with each replicate consisting of 20 flies.

Multivariate analysis was performed to identify the metabolic differences in both I and S selection regimes and to see how both the regimes respond to prick injury and bacterial infection. Fig 1 shows the experimental design, where four independent blocks were analyzed individually, and each of the four blocks had both I and S populations. Both the populations were further treated in three ways as mentioned in the introduction to have six different combinations of flies. An initial comparison of all six types of treatments namely I*u*, I*i*, I*s*, S*u*, S*i* and S*s* was done using the statistical method of PCA, an unsupervised method usually used to identify outliers and other patterns in the data. Fig 2(a) shows the PCA scores plot for all the six fly treatments with component 1 explaining 38.9% of the variation and component 2 explaining 25.4% of the variation. The figure shows one replicate sample of I*i* population to be located outside the 95% confidence area of the Hotellings T2 ellipse in the PCA score plot and thus being an outlier, was removed from further analysis. This was followed by analysis of all the six treatments using the supervised pattern recognition method of OPLS-DA. Fig 2(b) shows the cross-validated OPLS-DA scores plot, showing that irrespective of the type of treatment given, both I and S selection regimes are inherently different from each other. We then performed OPLS-DA analysis for different treatments within I and S regimes individually (S4 Fig). It can be seen from the predictive scores plot that individual treatments (*u*, *s* and *i*) are clearly separated out in both I and S regimes, showing that the treatment does affect the metabolome of both I and S regimes. Fig 3 shows the polar dendrogram plotted for all six treatments. As can be seen, the main node separates both I and S populations and further nodes separate different treatments within I and S regimes. This further confirms that both I and S populations have evolved to have different metabolomes from each other.

**Fig 4:**
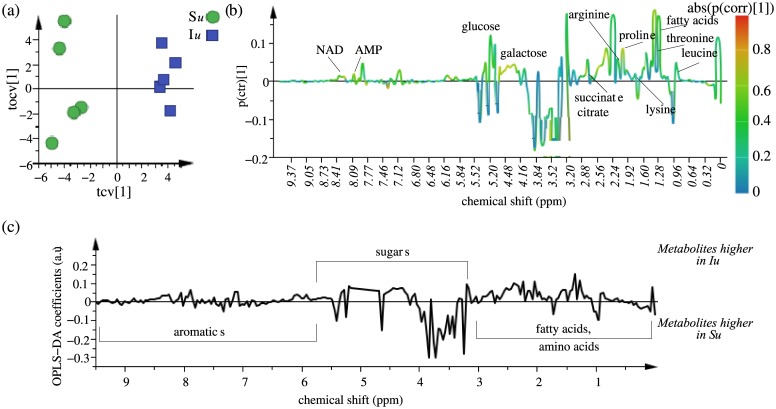
OPLS-DA analysis for S*u* and I*u* treatments. OPLS-DA (a) score plot and (b) loading plot derived from ^1^H NMR spectra of S*u* and I*u* treatments, with the section above 0 in the loading plot representing metabolites higher in the I*u* treatment and the section below 0 in the loading plot representing metabolites higher in the S*u* treatment and (c) S-line plot visualizing differences between I*u* and S*u* populations.

Since this initial comparison includes the differences both due to selection and due to treatments of prick injury and bacterial infection, we went on to individually compare the different fly treatments. In order to identify the metabolites responsible for differentiating I and S selection regimes, which have evolved over the course of several generations, we compared the unhandled control fly treatments (I*u* and S*u)*. We used the OPLS-DA statistical approach on 1D NMR spectral data aligned using the Icoshift algorithm [21, 27]. OPLS-DA maximizes the covariance between the measured data and the response variable (NMR peak intensities and predictive classifications respectively). Fig 4(a) shows the cross-validated OPLS-DA scores plot for comparison between I*u* and S*u* treatments, with one predictive component and one orthogonal component showing a clear separation between the I*u* and S*u* treatments. The loadings plot shows the significant metabolites responsible for separation between both the populations (Fig 4(b)). The significant metabolites were confirmed by first preselecting them using the VIP score parameter (VIP > 1), followed by identifying the metabolites from the linear S-plot (abs(p(corr) [1]) > 0.6) (Fig 4(c)). The variance explained *R*^*2*^ and the variance predicted *Q*^*2*^ by the modelare given in Table 1. The model therefore was an effective model and showed good predictive accuracy (S5 Fig). Testing with CV-ANOVA with α-value < 0.05 and validating with a permutation test (p-value < 0.05) also showed the model to be statistically significant and robust in nature. We further used hierarchical cluster analysis (HCA) to look for natural groupings in both the populations (S5 Fig). S5 Fig shows the tree dendrogram generated using HCA, summarizing the variation present in the dataset. As can be seen from the figure, individual replicates within the treatment were fairly similar to each other whereas both the I*u* and S*u* treatments were clearly separated from each other, indicating the predominant role of selection rather than genetic drift as a primary cause of evolutionary differentiation between the I and S regimes. The metabolite peak concentrations of fatty acids, organic acids (succinate and citrate), sugars (glucose and galactose), amino acids (leucine, proline, threonine, lysine and arginine), NAD and AMP were found to differ significantly in both the treatments and were found to be present in higher quantities in I*u* treatment as compared to S*u* treatment.

**Table 1:** Summary of parameters for assessing model quality.

Comparison	No. of components	R2X (cum)	R2Y (cum)	Q2Y (cum)
I*u*-S*u*	1+2	0.764	0.937	0.957
I*s*-I*u*	1+3	0.728	0.971	0.827
S*s*-S*u*	1+2	0.792	0.836	0.901
I*i*-I*u*	1+2	0.985	0.911	0.912
S*i*-S*u*	1+2	0.831	0.881	0.934

**Fig 5:**
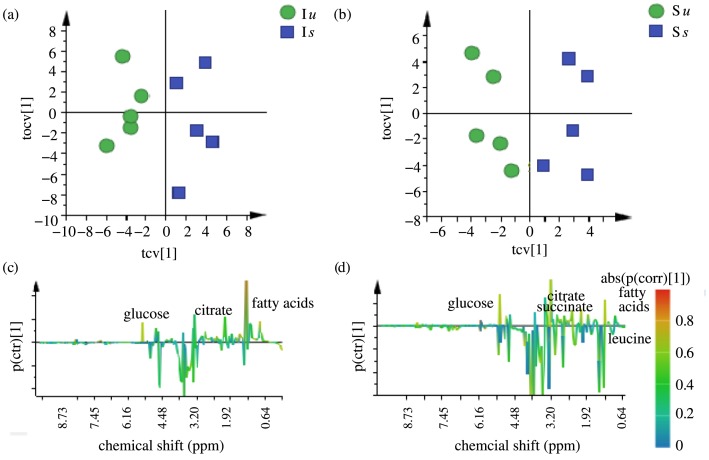
OPLS-DA analysis to see the effect of prick injury. (a) OPLS-DA score plot for I*s*-I*u* comparison, (b) OPLS-DA score plot for S*s*-S*u* comparison, (c) S-line plot for I*s*-I*u* comparison and (d) S-line plot for S*s*-S*u* comparison.

The above comparison identified the metabolites that get altered due to selection. In order to identify the metabolites that are significantly altered due to prick injury within the I and S lines, we compared the I*s* treated population with I*u* population and S*s* treated population with S*u* population. Fig 5(a) shows the cross-validated OPLS-DA scores plot for I*s*-I*u* comparison and Fig 5(b) shows the cross-validated OPLS-DA scores plot for S*s*-S*u* comparison. As can be seen from the figure, both I*s* and S*s* populations are clearly separated from I*u* and S*u* populations respectively along the predictive component. Fig 5(c) and 5(d) shows the respective S-line plots giving metabolites responding to prick injury within both the I and S lines. The model was validated using CV-ANOVA and permutation test as before. The variance explained by the models and the variance predicted are given in Table 1, showing that both the models were effective and had good predictive accuracy. For the I regime, the metabolite levels of fatty acids, glucose and citrate were found to differ significantly between the I*s* and I*u* populations and for the S regime, the levels of fatty acids, glucose, citrate, succinate and leucine were found to differ significantly between S*s* and S*u* populations, showing that these metabolites get affected during prick injury.

**Fig 6:**
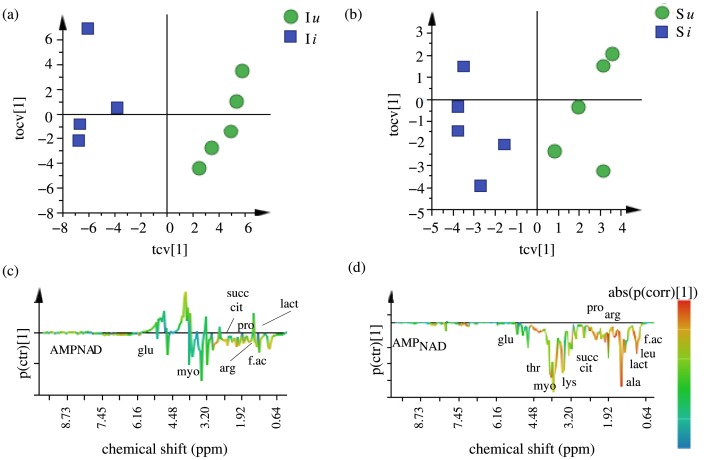
OPLS-DA analysis to see the effect of bacterial infection. (a) OPLS-DA score plot for I*i*-I*u* comparison, (b) OPLS-DA score plot for S*i*-S*u* comparison, (c) S-line plot for I*i*-I*u* comparison and (d) S-line plot for S*i*-S*u* comparison.

We then compared the I*i* treated population with I*u* population and S*i* treated population with S*u* population, to identify the metabolites that get affected by bacterial infection. Fig 6(a) and 6(b) shows the cross-validated OPLS-DA scores plots for I*i*-I*u* and S*i*-S*u* comparisons respectively. As can be seen from the figure, both I*i* and S*i* populations are clearly separated from I*u* and S*u* populations respectively along the predictive component. Fig 6(c) and 6(d) shows the respective S-line plots giving metabolites responding to bacterial infection within both the I and S lines. The model was again validated using CV-ANOVA and permutation test as before. Table 1 lists the variance explained and the variance predicted by the models, showing that both the models were effective and had good predictive accuracy. For the I regime, the metabolite levels of fatty acids, glucose, citrate, succinate, lactate, proline, arginine, myoinositol, NAD and AMP were found to differ significantly between the I*i* and I*u* populations and for the S regime, the levels of fatty acids, glucose, citrate, succinate, lactate, leucine, proline, lysine, arginine, alanine, threonine, myoinositol, NAD and AMP were found to differ significantly between S*i* and S*u* populations, showing that these metabolites get affected during bacterial infection.

**Fig 7:**
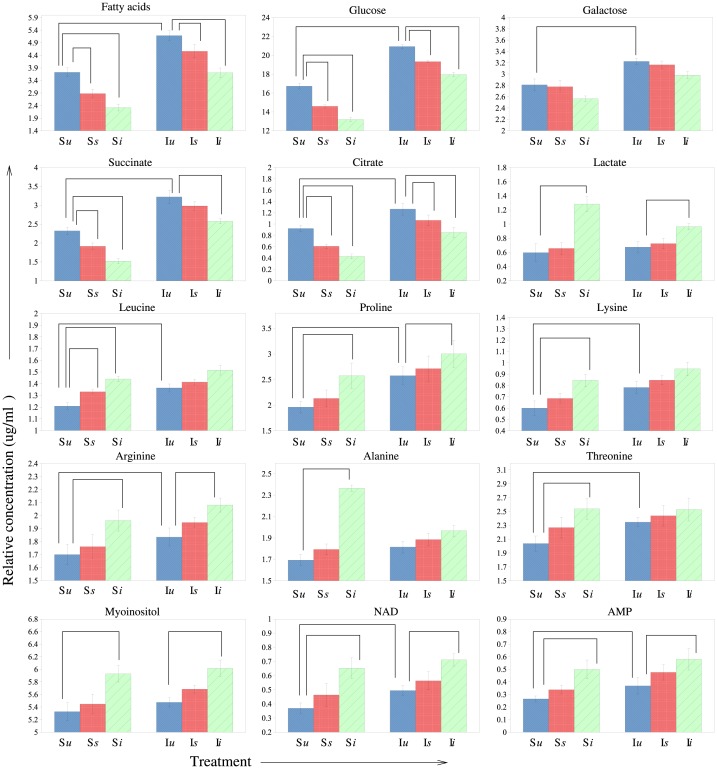
Plots of relative concentrations (mean ± SE) of significant metabolites in all the I and S treatments. X-axis shows the treatment given (untreated *u*, sham infected *s* and bacterial infection *i*) and Y-axis shows the relative concentrations of the metabolites. Comparisons included: I*u*-S*u* (selection), I*s*-I*u* and S*s*-S*u* (prick injury) and I*i*-I*u* and S*i*-S*u* (infection).

Univariate statistical analysis was also performed on all the variables identified in all the above pair wise comparisons for statistical significance using a t-test. A multiple hypothesis test correction method of Benjamini-Hochberg (BH) was applied to confirm the statistical significance of the identified metabolites. Concentration values of these significant metabolites in all the treatments are listed in S2 Table and shown as bar graph plots in Fig 7, with the BH corrected p-values for all the comparisons listed in Table 2.

**Table 2:** Metabolites, their chemical shift values and BH corrected p-values for all pair wise comparisons, with significant metabolites (p < 0.05) for that particular comparison marked in bold. ↑ (up) or ↓ (down) arrows indicate significant increase or decrease in concentration of metabolites respectively, in first treatment as compared to second treatment for each pair-wise comparison.

Metabolite	Peak (in ppm)	p-value (for pair-wise comparisons)						
		I*u/Su*	Is*/*I*u*	S*s/*S*u*	I*i/*I*u*	S*i/*S*u*	I*i/*I*s*	S*i/*S*s*
Fatty acids	1.27–1.31	**< 0.001↑**	**0.007↓**	**< 0.001↓**	**0.009↓**	**< 0.001↓**	**0.036↓**	**0.015↓**
Succinate	2.41–2.42	**0.032↑**	0.174	**0.046↓**	**0.014↓**	**0.019↓**	**0.047↓**	**0.009↓**
Citrate	2.53–2.55	**0.034↑**	**0.041↓**	**0.036↓**	**0.032↓**	**0.027↓**	**0.037↓**	**0.024↓**
Glucose	4.62–4.65	**0.014↑**	**0.036↓**	**0.009↓**	**0.031↓**	**0.018↓**	**0.033↓**	**0.038↓**
Galactose	4.07–4.08	**0.047↑**	0.946	0.913	0.088	0.287	0.149	0.480
Leucine	0.95–0.97	**0.035↑**	0.064	**0.039↑**	0.073	**0.029↑**	0.574	**0.036↑**
Proline	2.33–2.38	**0.028↑**	0.721	0.826	**0.042↑**	**0.032↑**	**0.017↑**	**0.004↑**
Threonine	4.24–4.28	**0.042↑**	0.825	0.723	0.063	**0.038↑**	0.382	**0.045↑**
Lysine	3.00–3.02	**0.035↑**	0.634	0.638	0.053	**0.035↑**	0.274	**0.040↑**
Arginine	1.60–1.75	**0.028↑**	0.058	0.063	**0.025↑**	**0.012↑**	**0.029↑**	**0.007↑**
Alanine	1.46–1.50	0.083	0.953	0.631	0.572	**0.018↑**	0.548	**0.017↑**
Myoinositol	4.05–4.07	0.642	0.713	0.724	**0.010↑**	**0.034↑**	**0.031↑**	**0.011↑**
Lactate	1.32–1.33	0.837	0.806	0.367	**0.028↑**	**0.037↑**	**0.029↑**	**0.006↑**
NAD	8.44–8.45	**0.046↑**	0.057	0.061	**0.019↑**	**0.006↑**	**0.038↑**	**0.013↑**
AMP	8.59–8.60	**0.037↑**	0.478	0.389	**0.004↑**	**0.035↑**	**0.041↑**	**0.037↑**

To confirm that the metabolic changes in the I and S regimes due to bacterial infection are indeed due to infection and not due to prick injury, we also compared I*i* treatment with I*s* treatment and S*i* treatment with S*s* treatment. S6 Fig shows OPLS-DA scores plot for I*i*-I*s* and S*i*-S*s* comparisons, showing clear separation between I*i*-I*s* and S*i*-S*s* treatments respectively. BH corrected p-values of the significant metabolites (identified using S-line plot) as listed in Table 2 show that the same set of metabolites contributes to significant differences between I*i*-I*s* comparison as identified for I*i*-I*u* comparison and between S*i*-S*s* comparison as identified for S*i-*S*u* comparison.

We also compared different selection lines for the same treatment type to see the effect of selection in the presence of treatment. S7 and S8 Figs shows OPLS-DA analysis for I*s*-S*s* (prick injury) and I*i*-S*i* (bacterial infection) comparisons respectively, showing clear separation between I*s*-S*s* treatments and between I*i*-S*i* treatments and confirming that both I and S selection lines remain different for the same treatment.

Univariate analysis was also performed on all the identified metabolites (a total of 37 metabolites whose peaks could be confidently assigned and quantified). Using a two-way ANOVA approach, we tested the effects of selection, treatments and their interactions on each metabolite and confirmed a robust metabolic response to both selection and treatments (S3 Table). Selection regime was responsible for the major metabolic response with 19 metabolites showing a significant effect of selection by having large F ratios and significant Q-values < 0.05. A total of 11 metabolites showed significant response to treatment, with many of them overlapping with selection as well. 5 metabolites responded differently to treatments between immune and sham selected populations (showing the effect of interaction between treatments and selection), with immune population showing less changes in the levels of these metabolites as compared to sham population which showed more changes in the levels of these metabolites upon treatment (Fig 7). The remaining 14 metabolites showed no effect of either selection or treatment. Most of the metabolites identified using univariate analysis and multivariate analysis overlap validating the results from both the methods.

## Discussion

### Impact of selection on metabolic profiles

Comparison between the un-infected selected (I*u*) and control (S*u*) populations clearly shows that the metabolome of the selected populations has evolved to be different from that of the control populations even under un-infected conditions (Figs 4 and 7). The selected population I*u*has evolved to have significantly higher concentrations of fatty acids, carbohydrates (glucose, galactose), organic acids (citrate, succinate), amino acids (leucine, proline, lysine, arginine, threonine), NAD and AMP as compared to the control population S*u*. Selection pressures have previously been reported to alter the metabolome of *Drosophila* [23, 28]. Our results also highlight the role played by selection in altering the metabolome of fruitfly *Drosophila*. It should be emphasized here that the relative concentrations (normalized to total intensity) of metabolites identified to be significantly different in selected and control populations are a result of the flux of a large number of inter-related metabolic processes so it is difficult to discern the exact metabolic pathways getting affected by selection and further investigations are thus needed in order to achieve a better understanding of the dynamics of metabolites and their relation and response to selection.

### Impact of prick injury on metabolic profiles

Prick injury alters the metabolic profiles of both the selected and control regimes (Figs 5 and 7). However, the same class of metabolites (fatty acids, carbohydrates and organic acids) is affected in both the populations, showing a similarity in response, which is seen in the form of decrease in fatty acids, carbohydrates and organic acids. This shows that there is no change in the response to prick injury due to selection as both I and S populations respond in a similar fashion. It should be noted that even though both the populations respond in a similar manner, the relative concentrations of all the prick responsive metabolites are still significantly higher in selected populations (I*s*) than control populations (S*s*) (Fig 7, S6 Fig).

### Impact of infection on metabolic profiles

Upon infection, the metabolic profiles get affected at the levels of fatty acids, carbohydrates, organic acids, amino acids and energy related metabolites like NAD, AMP in both selected and control populations (Figs 6 and 7). Again the direction of response is the same for both selected and control populations in the sense that the change in a given metabolite post infection are similar in direction in both selected and control populations. However as can be seen from Fig 7, the change in concentration in case of many significant metabolites (lactate, leucine, proline, lysine, arginine, alanine, threonine) is less severe in case of selected populations as compared to control populations, which shows more sharp change in concentrations upon infection. Also, the relative concentrations of these metabolites are still significantly higher in I*i* populations than S*i* populations (Fig 7, S7 Fig). Two inferences can be drawn from all of the above comparisons- (1) the selected I populations continue to have higher concentrations of most of the significant metabolites with or without the treatments as compared to control populations and (2) the metabolome of the selected populations seems to be slightly more robust to changes due to treatments than control populations, indicating a better maintenance of metabolic homeostasis during bacterial infection.

### Implications of changes in metabolite levels due to selection and treatments

With respect to certain metabolites, the immediate phenotypic response to infection/injury and evolutionary response to infection seem to be in line with each other. For example, the concentration of amino acids like proline, arginine, lysine, NAD, AMP, myoinositol increases with infection/injury in the control populations and the I populations have higher concentration of these metabolites even under un-infected conditions. However, looking at concentration changes in certain other metabolites during proximate infection gives an impression that evolutionary and immediate phenotypic responses push the metabolome in opposite directions. For example, concentration of fatty acids, sugars, organic acids decreases upon infection/injury in the control populations, yet the selected populations evolve to have higher levels of these metabolites. Such discordance between phenotypic and evolutionary responses has been documented extensively in flies [29]. Yet as we shall argue below, overall selection seems to push the whole metabolome towards being better prepared to fight infections.

In both the I and S regimes, post infection/injury, metabolites such as fatty acids, glucose, succinate and citrate that are typically associated with energy-related pathways, decline in concentration indicating that mounting a response to infection/injury is an energy intensive process (Fig 7, Table 2, S2 Table). Similar to our results of decrease in fatty acids and carbohydrates, a decrease in fatty acids has previously been reported during recovery phase following exposure to heat stress [14, 30] and during *V*.*cholerae* infection in drosophila [31] and decrease in both fatty acids and carbohydrates has been reported during *Listeria monocytogenes* infection [6] wherein energy metabolism has been shown to get affected. Increase in concentration of other metabolites like NAD, AMP, leucine and alanine (all associated with energy-yielding pathways) post infection/injury also indicates increased energy requirements (Fig 7, Table 2, S2 Table). Thus, given that energy requirements are high post injury/infection, one would expect our selected populations to be better prepared to fight infections in terms of having higher concentrations of energy rich metabolites (fatty acids, sugars, organic acids, NAD, AMP, ceratin amino acids) even under un-infected conditions. In line with this expectation, we find higher concentrations of fatty acids, sugars, organic acids, NAD and AMP in I populations even under un-infected conditions (Fig 7, Table 2, S2 Table).

The concentration of many amino acids (proline, lysine, arginine) increases post infection/injury in both the I and S populations. Amino acids are components of antimicrobial peptides which are an important component of *Drosophila* immunity [32]. Hence, their increase post infection is expected. Therefore, it is also expected that the selected populations may evolve to have higher concentration of amino acids even under un-infected conditions to be better prepared to handle infection. This is again borne out by the increased concentration of these amino acids like proline, arginine, threonine in the un-infected I populations. Similarly, some other amino acids like lysine which have a role in clot formation [33] undergo an increase during infection in both the populations indicating a possibility of its role in clot formation during infection/injury in our populations and are also present in higher quantities in selected populations.

Lactate, an indicator of anaerobic metabolism during stressful conditions [34], increases at a greater rate in the control populations post infection, indicating a possibility of higher stress and energy requirements in control populations following infection. However, the increase in lactate in the selected I populations is much lower, indicating that they have probably evolved ways to handle infection related stress. This is further supported by the fact that myoinositol which functions as messenger in signal transduction and helps in coping with stress [35–38] was also present in higher concentration in the selected populations.

It is important to note that while both I and S regime flies show similar pattern with most metabolites post infection/injury (for example amino acids like proline, arginine, molecules like NAD, AMP, myoinositol increase in both I and S post infection/injury while fatty acids, sugars like glucose, organic acids like succinate, citrate decrease in both I and S post infection/injury), the magnitude of change is not always similar in I and S populations, indicating that probably at least a part of the response to infection/injury has evolved to be different between S and I populations.

Thus, in general, the overall push of selection seems to be towards better preparing the metabolome to withstand and fight the harmful effects of infection.

## Materials and methods

### Fly stock

The fly stocks have been described in detail in [39]. The selected populations were derived from a laboratory adapted population—BRB (Blue Ridge Baseline). BRB is an outbred population started from 19 isofemale lines caught from the wild in Blue Ridge, USA. After about 10 generations, four replicate populations of BRB_(1–4)_ were created. These independent replicate populations are maintained on a 14-day discrete generation cycle, 12:12 light:dark regime, 25°C temperature and 60–70% relative humidity. Every generation, about 70 eggs were transferred to 6 mL of standard banana-jaggery food, in 8 dram vials. 40 such vials were set up per population. On the 12th day post egg collection, flies were transferred to a fly cage (25 cm length × 20 cm width × 15 cm height) with standard banana-jaggery food and *ad libitum* live yeast paste. On the 14th day post egg collection, a food plate was provided for 18 hours for oviposition. After 18 hours, eggs were collected at a density of 70 eggs per vial. The adult population size of each replicate was maintained close to 2500 individuals.

### Selection regime

The derivation and maintenance of the selection regime has been described in detail in [40]. Briefly, a total of three selection regimes were created from each BRB population- I (Infected with pathogen), S (Sham Infected) and U (Unhandled control). For these NMR experiments, two regimes were used–I (Infected with pathogen) and S (Sham Infected) whereas U (Unhandled control) was omitted. With an aim to identify metabolites responsive to bacterial infection, I regime was compared with S regime (as its control) to negate the effect of prick injury from bacterial infection. Thus we had four I populations I_(1–4)_ and four S populations S_(1–4)_. Since I and S populations bearing the same numerical subscript were derived from the same BRB population, they were more closely related to each other than to any other population. For example, I_1_ and S_1_were derived from BRB_1_ and were more closely related to each other than to I_2_ or S_2_. These populations bearing the same numerical subscript were treated as Random Blocks in the statistical analysis. Thus, I_1_ and S_1_ constituted Block 1 and so on. In the I populations, on the 12th day post egg collection, 150 males and 150 females were infected with a bacterial suspension of a gram-negative pathogen *Pseudomonas entomophila*. In the S populations, 100 males and 100 females from S were pricked with a needle dipped in a 10 mM MgSO4 solution. These flies were then maintained in cages (14cm length × 16cm width × 13cm height) for four days during which ~40% of the flies in the I population died due to infection. On 16^th^ day, food plate was given for 18 hours for oviposition. After 18 hrs of egg laying, eggs were collected at a density of 70 eggs per vial, with 10 such vials for each population. All the populations were maintained on a standard banana-jaggery diet in a 12:12 light:dark regime, 25°C temperature and 60–70% relative humidity. Each Block containing two populations (I and S) was handled independently on different days. Adult numbers of the S populations were scaled according to the percentage survivorship in the I populations. The number of flies in S was also increased when the survivorship in the I population changed, most likely due to selection.

### Bacterial stock

*Pseudomonas entomophila*(strain L48 (Pe)) was procured from Dr Brian Lazarro’s lab at Cornell University, New York. The bacteriawas cultured in LB medium at 27°C. For infection during selection, an overnight culture of bacteria was subcultured by diluting it 1000 fold. Bacterial culture of optical density (OD) 1 at 600 nm was pelleted down and suspended in equal volume of 10 mM MgSO_4_ to obtain a final OD 1. This bacterial suspension was used for infections. OD of the bacterial culture was subsequently increased whenever survivorship in I populations reached above 50%. Increase in OD was not required until five generations of selection. Post five generations of selection, OD was increased every 2–3 generations in small increments. For infection during treatment, heat killed bacteria was used instead of live pathogen. Final OD of the pelleted down bacteria was set at 1.5. Bacterial suspension was then heated at 92°C for half an hour. It was then cooled down and used for infecting the standardized flies. It was confirmed that the bacteria were killed by the treatment, by wiping a swab containing bacterial suspension on the surface of LB agar containing plate and looking for the colonies of bacteria after 24 hours. The absence of any bacterial colony confirmed that the bacteria had been heat killed [39].

### Standardization of flies

To equalize non-genetic parental effects, we passed all the I and S populations through one generation of common rearing during which they were neither infected nor pricked. We refer to these flies as standardized flies.

### Experimental set-up

All NMR experiments were done on the flies after 40 generations of selection. Flies from both the regimes were transferred to cages on the 12th day and given live yeast paste for two days. We then collected eggs from the standardized flies from the I and S populations at a standard density and maintained at standard laboratory conditions (see above). On the 10^th^ day post egg collection, when the flies had just begun to eclose, virgin female flies were collected by sorting the flies using light CO_2_ anesthesia and used for NMR experiments. Flies were handled at a density of 20 females per vial. On the 12^th^ day post egg collection (when the flies were 2–3 days old as adults), flies from the both the I and S populations were randomly assigned to one of the three treatments: (i) infected by pricking with a fine needle (Minutein pin 0.1 mm, Fine Science Tools, CA) dipped in heat-killed bacterial suspension (bacteria suspended in 10 mM MgSO_4_) in the thorax (labeled as I*i* and S*i* respectively), (ii) infected with a needle dipped in sterile 10 mM MgSO_4_ (labeled as I*s* and S*s* respectively) and (iii) unhandled controls (labeled as I*u* and S*u* respectively). Treatment (i) in both I and S populations served to see the changes in the metabolome due to immune response to bacterial infection whereas treatment (ii) served to see the changes in the metabolome due to prick injury. Treatment (iii) identified the basal differences in the metabolome of both the populations due to their different evolution. Each treatment had 5 replicates, with each replicate having 20 flies to make a total of 100 flies per treatment. There was no difference in body size between control and selected populations [40]. Flies in the vials were flash-frozen, 6 hours after the treatment, by immersing the vials in liquid nitrogen and then transferred to pre-labeled microfuges for NMR experiments.

### NMR sample preparation

Flies from each treatment were mechanically homogenized using a battery run homogenizer in 300 *μ*L of ice-cold acetonitrile (50%) and centrifuged at 12000 rpm for 10 min at 4°C. The supernatant was transferred to new, pre-weighed tubes, lyophilized and stored at −80°C until the NMR experiments were performed. Immediately before the NMR measurements, the samples were rehydrated in 500 *μ*L of D2O, containing 0.5mg/ml of an NMR reference 3-(trimethylsilyl)-propionicacid-D4 sodium salt (TMSP),and then transferred to 5mm NMR tubes.

### NMR spectroscopy and data analysis

High-resolution NMR spectra were recorded at 298 K on a BrukerBiospin 400 Avance-III spectrometer operating at a ^1^H frequency of 400 MHz and equipped with a 5 mm BBO rf probehead. Three different kinds of 1D ^1^H NMR spectra were acquired: (i) One-dimensional spectra with water presaturation achieved by cw irradiation applied during the relaxation delay to suppress the residual HOD signal. (ii) One-dimensional spectra with water suppression using the NOESY presaturation sequence with a mixing time of 10 ms and (iii) a water-suppressed Car-Purcell-Meiboom-Gill (CPMG) spin-echo pulse sequence optimized for a spin echo delay time *t* of 300 *μ*s (*n* = 400) and a total spin-spin relaxation delay (2*nt*) time of 240 ms to achieve attenuation of fast-relaxing broad signals from larger molecules. The proton 1D spectra were collected with a 90° pulse width of 12.25 *μ*s, a relaxation delay of 1s, 32 scans, 64 K data points and a spectral width of 12 ppm. Data were zero-filled by a factor of 2 and the free induction decays (FIDs) were multiplied by an exponential weighting function equivalent to a line broadening of 1 Hz prior to Fourier transformation. Automatic phasing and polynomial baseline correction was performed, and resonance peaks were referenced to the TMSP resonance at *δ* = 0.00 ppm. For resonance assignment and metabolite identification, a set of two-dimensional NMR spectra were recorded, including correlation spectroscopy (COSY), total correlation spectroscopy (TOCSY), nuclear Overhauser effect spectroscopy (NOESY), and heteronuclear single quantum and multiple quantum coherence spectroscopy (HSQC, HMQC). 2D ^1^H-^13^C heteronuclear spectra were obtained with a spectral width of 12 ppm and 220 ppm in the proton and carbon dimensions respectively, with 1K data points, 16 scans, 256 t_1_ increments and a recycle delay of 2s. The 2D homonuclear COSY and TOCSY spectra were acquired with a spectral width of 12 ppm in both dimensions, with 1K data points, a recycle delay of 2s, 16 scans and 256 t_1_ increments.

### Metabolite identification

Metabolites were identified from peaks in the 1D proton NMR spectra and from proton-carbon coupling information obtained from 2D HSQC and HMQC experiments. Non-overlapped peak intensities that were identified as belonging to a single compound, were chosen for quantification. Peak integrals were compared to the integral of the NMR internal standard TMSP. Metabolite identification was confirmed by matching metabolite peaks with standard NMR spectral databases such as the Biological Magnetic Resonance Data Bank (BMRB) http://www.bmrb.wise.edu and the Madison Metabolomics Consortium Database (MMCD) http://mmcd.nmrfam.wise.edu.

### Statistical analysis

The entire range of resonances in the 1D ^1^H NMR spectra was used without binning for the statistical analysis. Spectral data were converted into ASCII format and aligned using the *Icoshift* algorithm [27]. The spectral region between *δ* = 4.6 − 4.8 ppm was excluded from the analysis, to remove errors due to the residual water signal. Data were normalized to the total area, to be able to compare spectra with possibly different signal-to-noise ratios.

Multivariate statistical analysis was performed using SIMCA 14.0 software (Umetrics Umea Sweden) and MetaboAnalyst 2.0 http://www.metaboanalyst.ca), a web-based platform for metabolomics data analysis [41]. The data were Pareto-scaled and initially analyzed using the unsupervised method of principal component analysis (PCA). Data points located outside the 95% confidence area of the Hotellings T2 ellipses in the PCA score plots were treated as outliers and excluded from further analysis. The performance of the PCA model was evaluated using the coefficient *Q*^*2*^ (using the 7-fold internal cross-validation method [42]) and the coefficient *R*^*2*^, defining the variance predicted and explained by the model, respectively. The data were further analyzed using the supervised pattern recognition method of orthogonal projections to latent structures-discriminant analysis (OPLS-DA), which maximizes the class discrimination. The loadings plot was used to identify significant metabolites responsible for maximum separation in the OPLS-DA scores plot and these metabolites were ranked according to their variable influence on projection (VIP) scores. VIP scores are weighted sums of squares of the OPLS-DA weights, which indicate the importance of the variable. The VIP score of the first principal component in the OPLS-DA model (VIP *>*1.0) was used to screen significantly different metabolites. This was followed by identifying the significant metabolites using the S-line plot which visualizes the covariance (p(ctr)) and correlation coefficient (abs(p(corr)) between the variables and the classification score in the model. The magnitude of covariance is difficult to interpret as it is scale dependent and influenced by the intensity of the signal with respect to the noise level and thus will likely indicate variables with large signal intensities. The correlation coefficient gives a linear indication of the strength of the correlation and is independent of the intensity of the variable so it is a better measure for the reliability of the variable in the classification process. Strongly discriminating variables have a clear covariance with a high absolute value for the correlation coefficient [43]. The quality of the model was evaluated by the *R*^*2*^ and *Q*^*2*^ values, defining the variance explained (indicating goodness of fit) and the variance predicted by the model, respectively. The performance of the OPLS-DA model was evaluated using CV-ANOVA (cross-validated ANOVA) [44], where *α*-value *<*0.05 was considered to be statistically significant. Permutation analysis was further performed on the best model, using 1000 permutation tests with a threshold *p* value of *<*0.05 indicating that none of the results are better than the original one [45]. Hierarchical cluster analysis (HCA) was performed to create a tree dendrogram using the complete linkage algorithm and the Euclidean distance between OPLS-DA scores. Fan (polar) dendrograms were also generated using a MATLAB script (http://www.mathworks.com/matlabcentral/fileexchange/21983-draw-a-polar-dendrogram), to better visualize the clustering. Univariate analysis was also performed to confirm the statistical significance of the metabolites identified. For all the two group comparisons, a t-test was performed on the ^1^H NMR peak integrals corresponding to the significant metabolites. Metabolites with VIP>1, S-line plot abs(p(corr)>0.6 and t-test p-value <0.05 were then selected. Multiple testing correction was also applied to adjust the individual p-value for each metabolite from the t-test using the Benjamini-Hochberg method [46] with the level of significance selected as 0.05. Two-way ANOVA was also performed on all the identified metabolites to test the effect of selection, treatment and their interaction on each metabolite using the statistical software package STATISTICA (StatSoft. Inc., Tulsa, Oklahoma).

## Conclusions

Our study is the first to show the evolution of the metabolome in response to selection for immunity. We show that the I regime selected for immunity is evolved to be better prepared to handle infection than the S regime, through alterations in lipid, carbohydrate and amino acid metabolisms. The NMR experiments provide tantalizing evidence for the important role played by the *Drosophila*metabolome along its evolutionary trajectory, in response to selection forincreased immunity.

## Supporting information

S1 Fig 1D 1H NMR spectra showing a few peaks of identified metabolites. (PDF)

S2 Fig 2D COSY NMR spectrum of *D*. *melanogaster* with peaks used for metabolite identification. (PDF)

S3 Fig 2D HSQC NMR spectrum of *D*. *melanogaster* with peaks used for metabolite identification. (PDF)

S4 Fig OPLS-DA analysis of treatments within I and S regimes (a) Predictive scores plot for I regime (b) Predictive scores plot for S regime. (PDF)

S5 Fig Evaluation of OPLS-DA model for I*u*-S*u* comparison and HCA for replicates within I*u* and S*u* population. (PDF)

S6 Fig OPLS-DA score plot of I*i*-I*s* and S*i-*S*s*. (PDF)

S7 Fig OPLS-DA analysis for I*s* and S*s* treatments. (PDF)

S8 Fig OPLS-DA analysis for I*i* and S*i* treatments. (PDF)

S9 Fig OPLS-DA score plot of all six populations of Block 1. (PDF)

S10 Fig OPLS-DA score plot of all six populations of Block 2. (PDF)

S11 Fig OPLS-DA score plot of all six populations of Block 4. (PDF)

S1 Table List of metabolites identified from 1D 1H and 2D NMR experiments. (PDF)

S2 Table Relative amounts of metabolites present in different treatments. (PDF)

S3 Table Two-way ANOVA with fixed factors of selection, treatment and their interaction for Block 3. (PDF)

S4 Table Two-way ANOVA with fixed factors of selection, treatment and their interaction for Block 1. (PDF)

S5 Table Two-way ANOVA with fixed factors of selection, treatment and their interaction for Block 2. (PDF)

S6 Table Two-way ANOVA with fixed factors of selection, treatment and their interaction for Block 4. (PDF)

S1 File Bruker format 1D ^1^H NMR raw data for Block 1. (ZIP)

S2 File Bruker format 1D ^1^H NMR raw data for Block 2. (ZIP)

S3 File Bruker format 1D ^1^H NMR raw data for Block 3. (ZIP)

S4 File Bruker format 1D ^1^H NMR raw data for Block 4. (ZIP)
