# Supplementary figures and images for: Evolution of the metabolome in response to selection for increased immunity in populations of Drosophila melanogaster
Source: PLoS One. 2017 Nov 17;12(11):e0188089. doi: 10.1371/journal.pone.0188089 (PMC5693281; doi:10.1371/journal.pone.0188089)

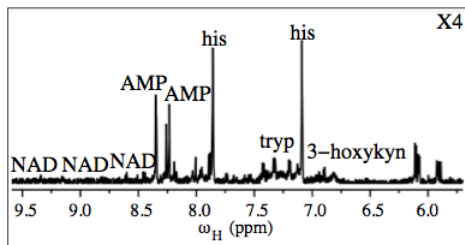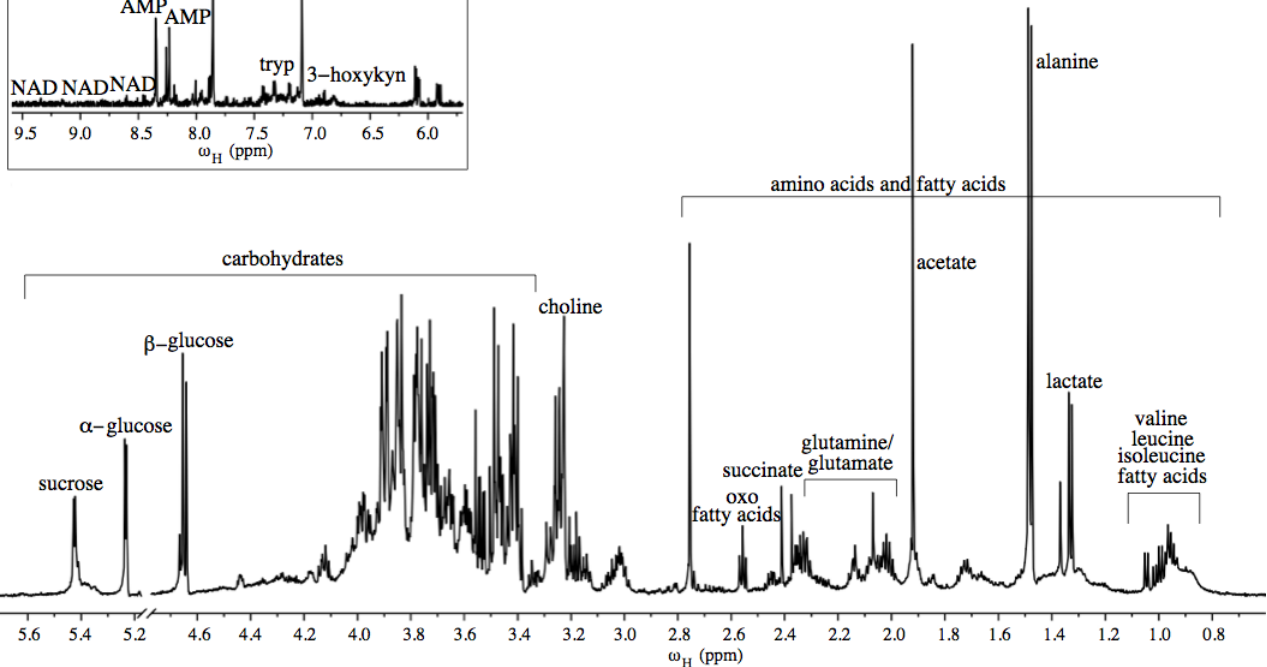

Supplement: S1 Fig — (PDF) [file pone.0188089.s001.pdf]

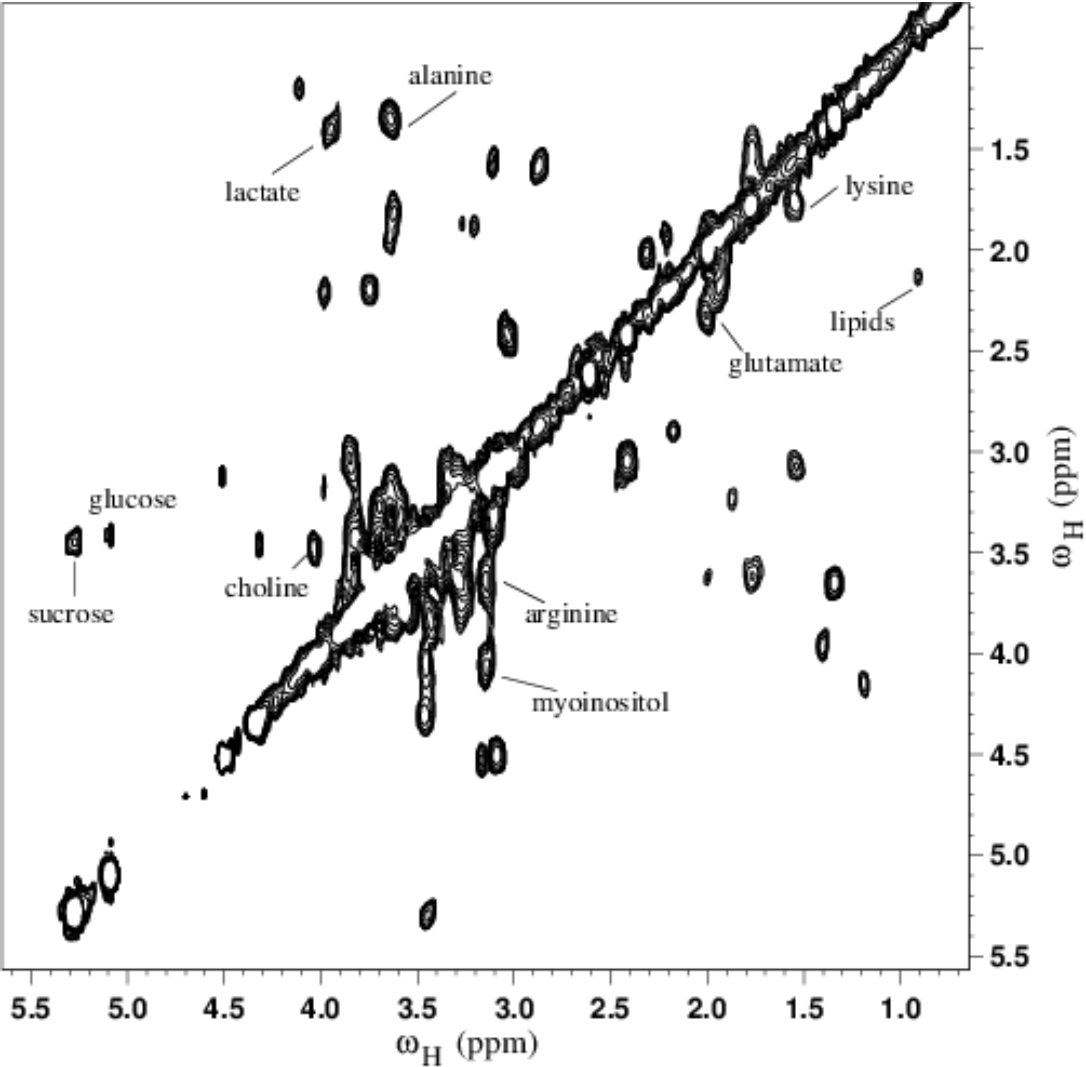

Supplement: S2 Fig — (PDF) [file pone.0188089.s002.pdf]

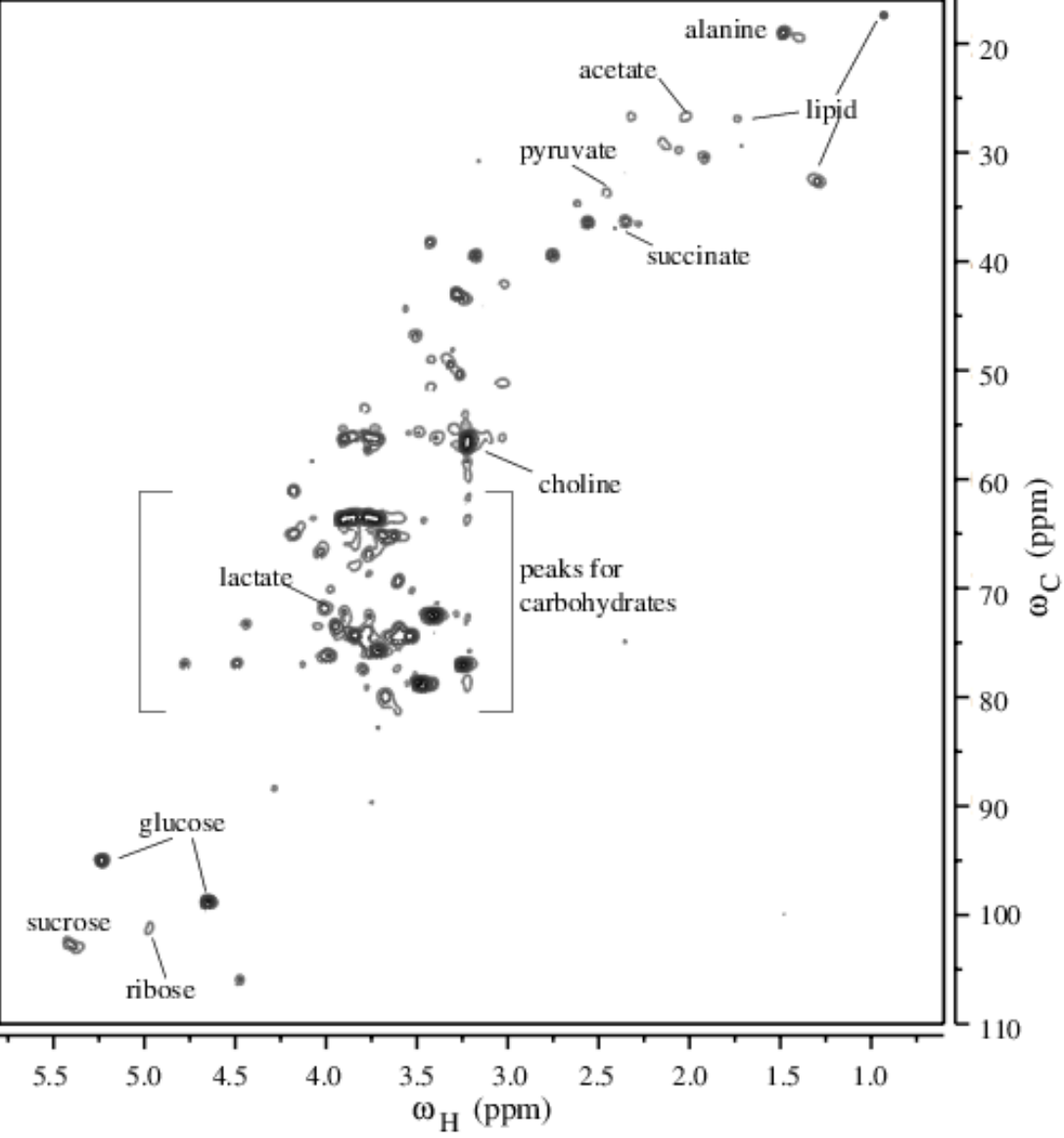

Supplement: S3 Fig — (PDF) [file pone.0188089.s003.pdf]

(a)

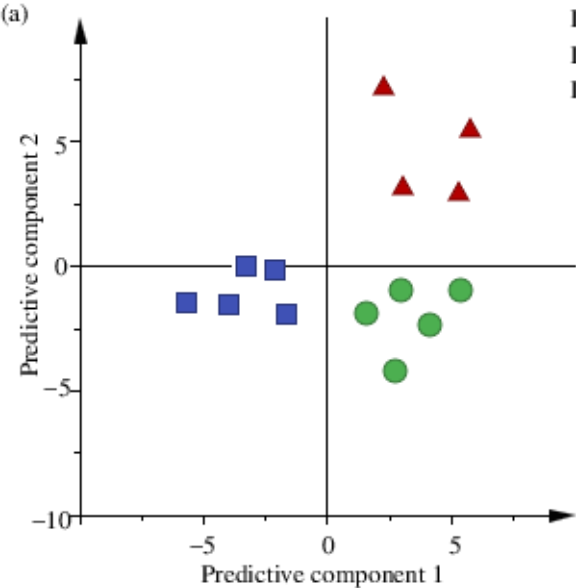

(b)

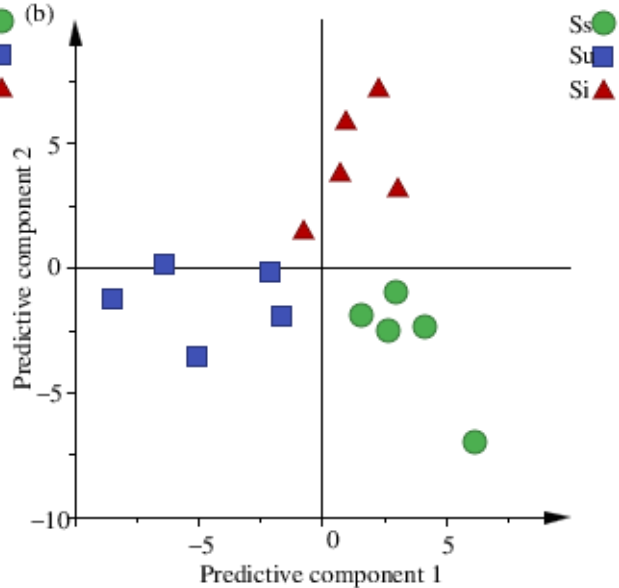

Supplement: S4 Fig — (PDF) [file pone.0188089.s004.pdf]

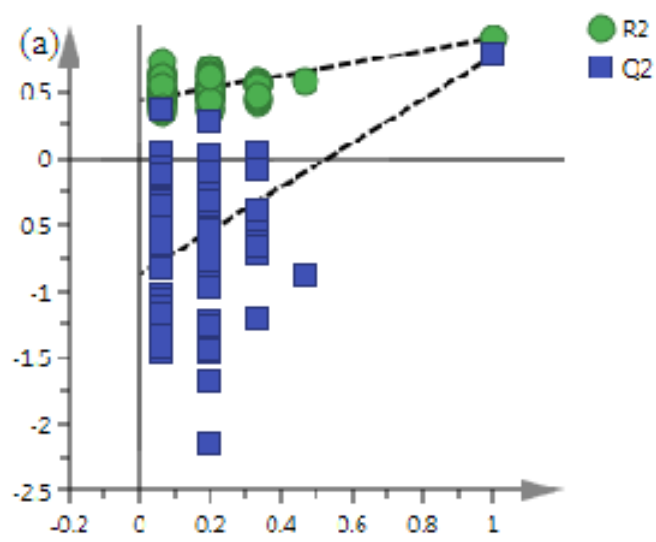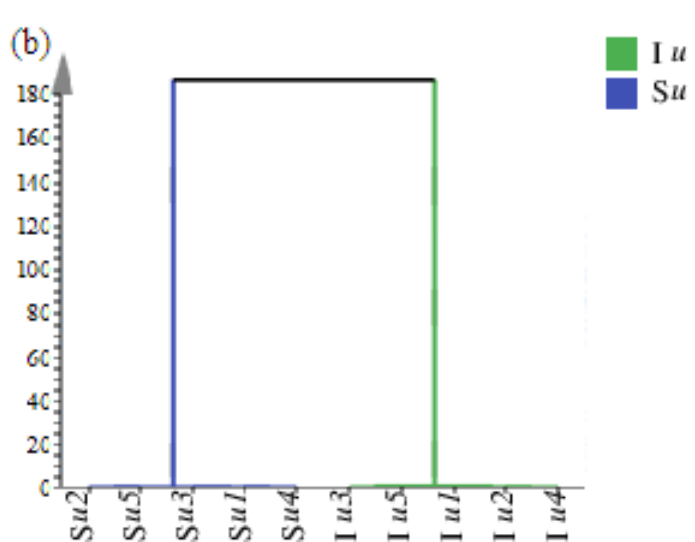

Supplement: S5 Fig — (PDF) [file pone.0188089.s005.pdf]

Scores Plot

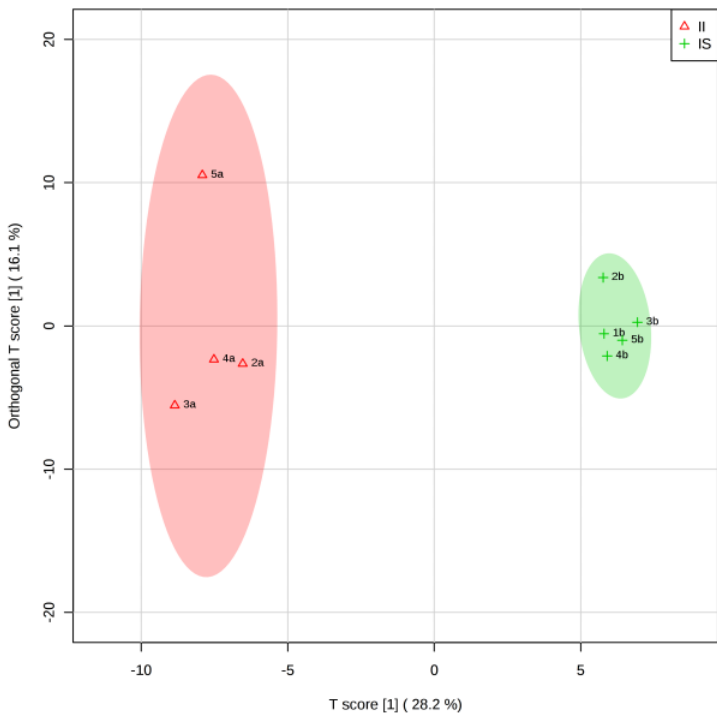

Scores Plot

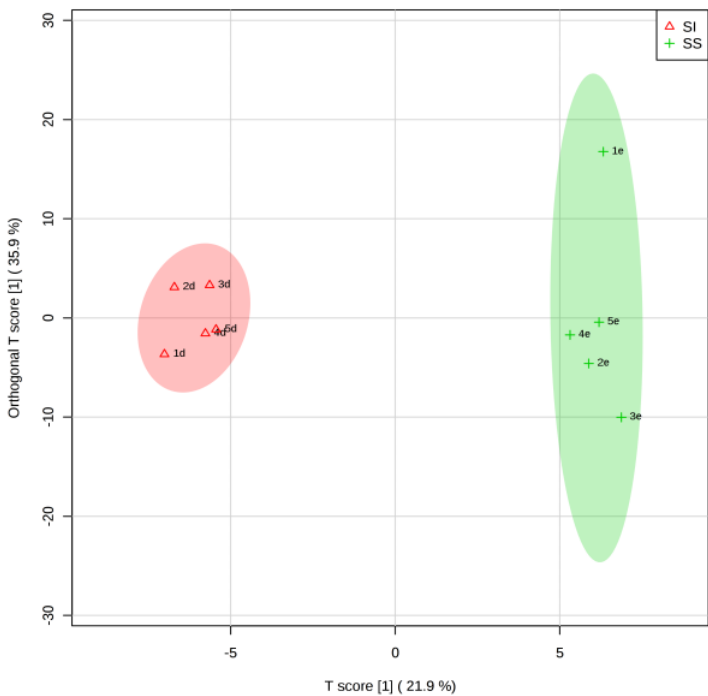

Supplement: S6 Fig — (PDF) [file pone.0188089.s006.pdf]

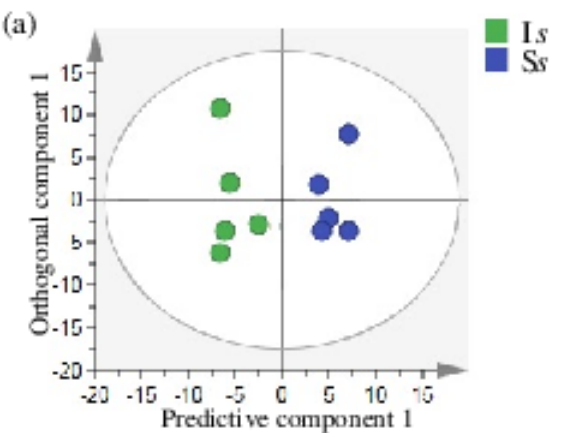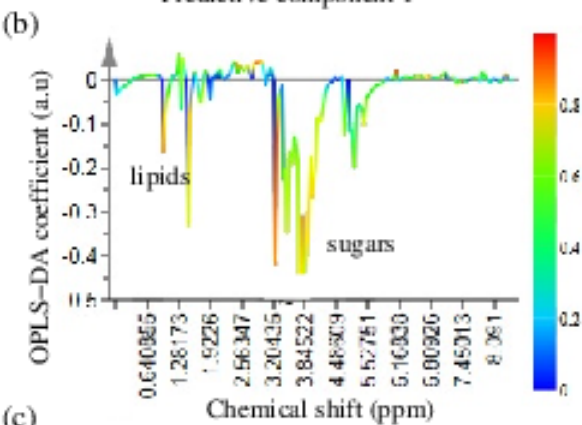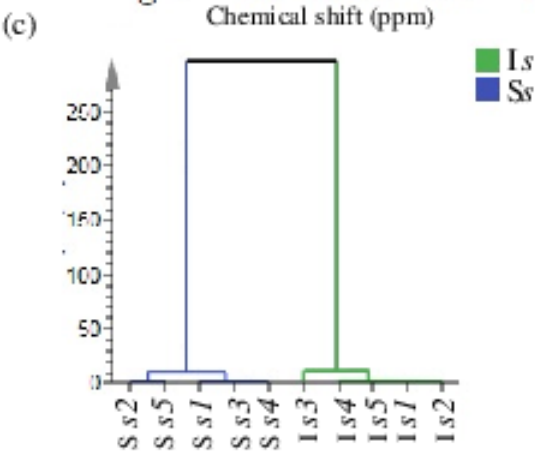

Supplement: S7 Fig — (PDF) [file pone.0188089.s007.pdf]

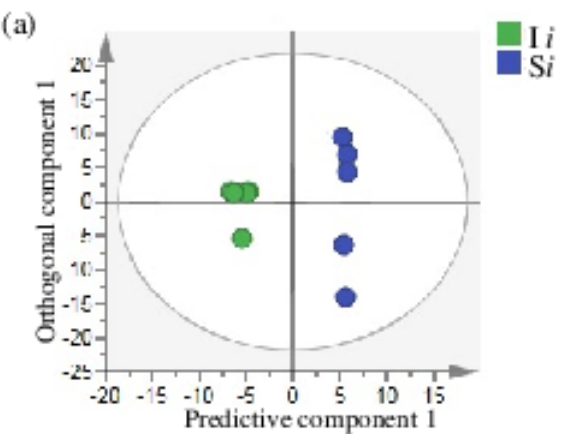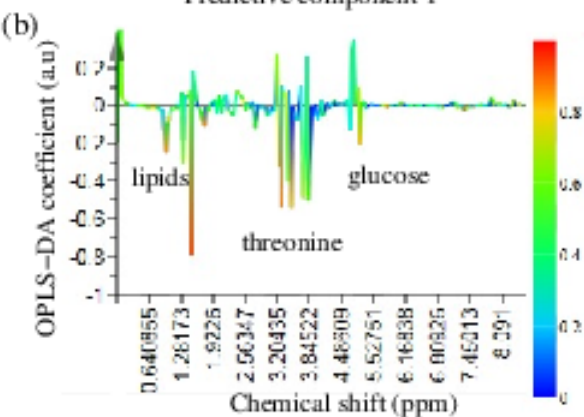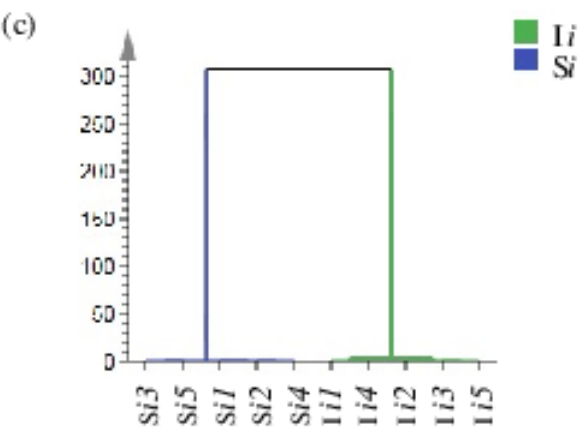

Supplement: S8 Fig — (PDF) [file pone.0188089.s008.pdf]

# Scores Plot

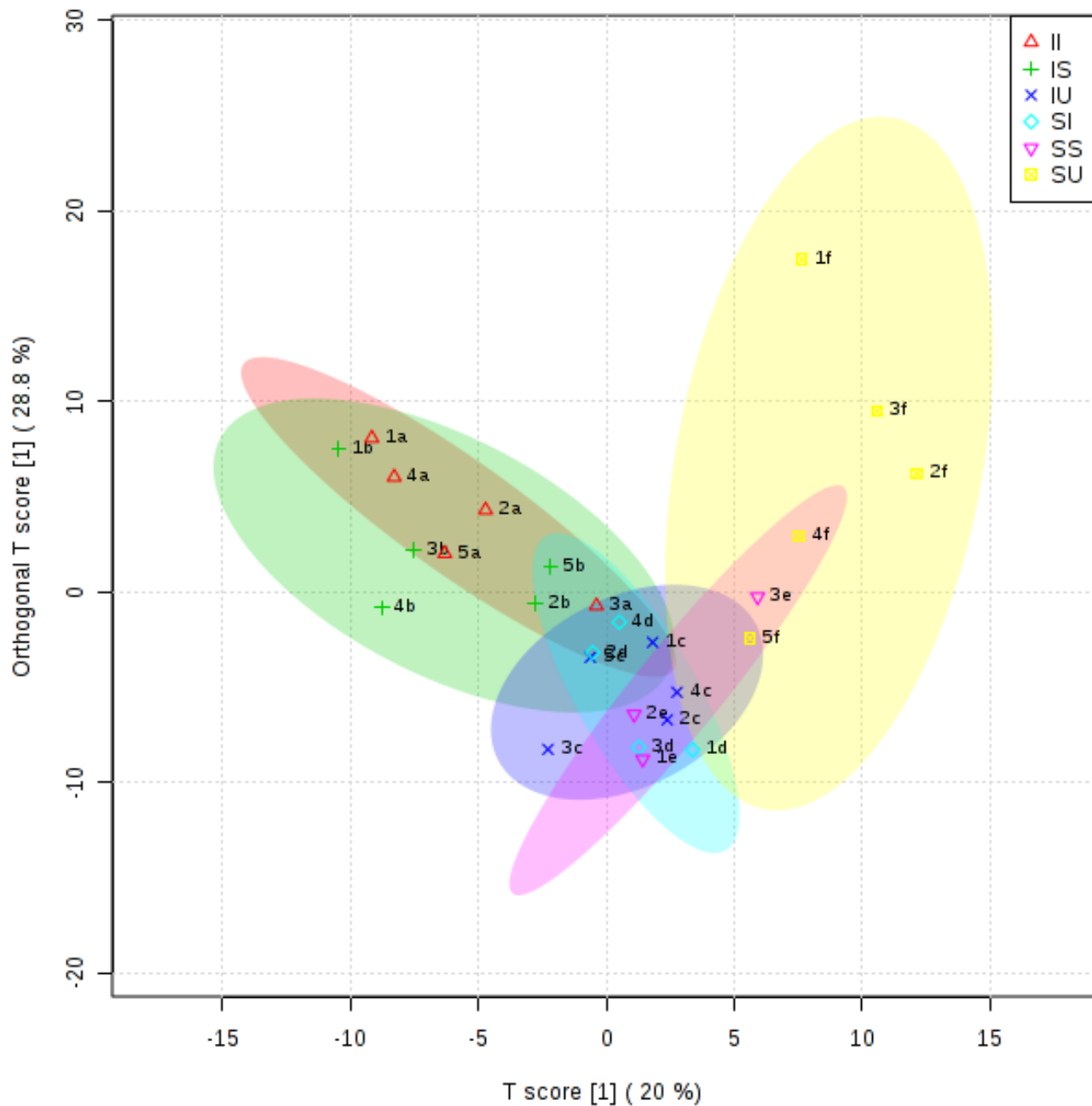

Supplement: S9 Fig — (PDF) [file pone.0188089.s009.pdf]

**Scores Plot**

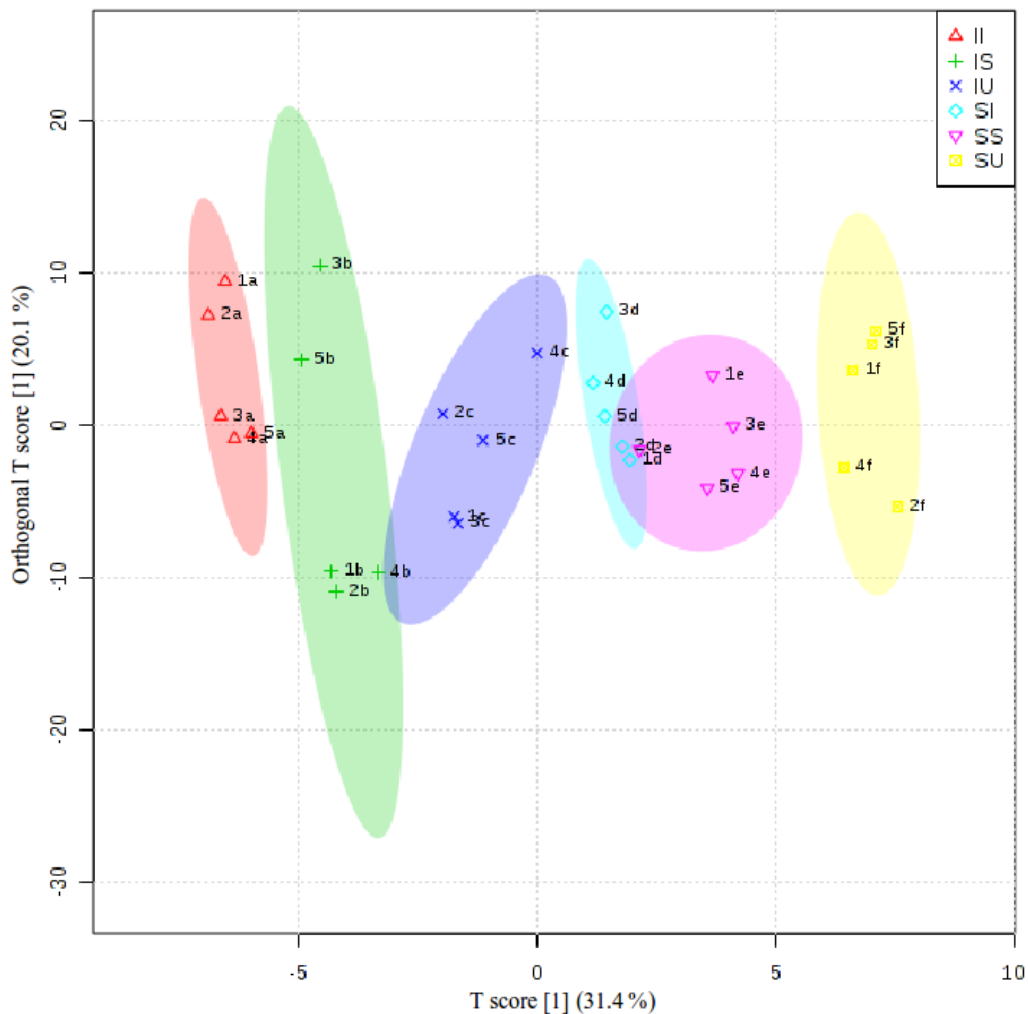

Supplement: S10 Fig — (PDF) [file pone.0188089.s010.pdf]

**Scores Plot**

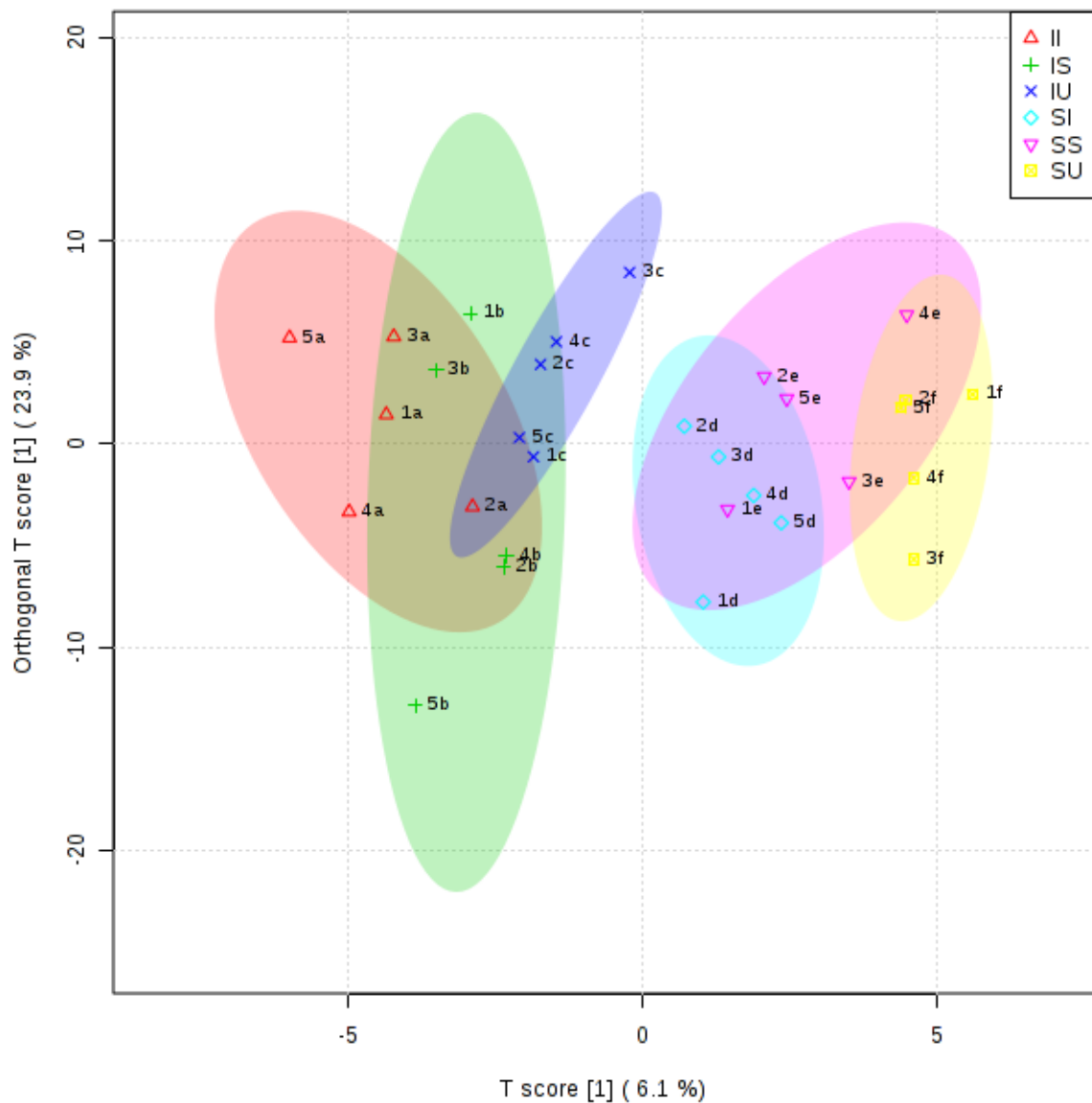

Supplement: S11 Fig — (PDF) [file pone.0188089.s011.pdf]
