# Supplementary material for: Evolution of the metabolome in response to selection for increased immunity in populations of Drosophila melanogaster
Source: PLoS One. 2017 Nov 17;12(11):e0188089. doi: 10.1371/journal.pone.0188089 (PMC5693281; doi:10.1371/journal.pone.0188089)
Supplement: S1 Table — (PDF) [file pone.0188089.s012.pdf]

Metabolite	Chemical shift in ppm (multiplicity, J in Hz)	Metabolite	Chemical shift in ppm (multiplicity, J in Hz)
**Sterols**	0.76 (s)	**Carbohydrates**	
**Lipids**		Ribose	2.21 (s)
terminal methyl group	0.89 (t,6.9)	Trehalose	3.82(m), 5.18 (d, 3.8)
-(CH_2)n	1.27 (m)	Galactose	3.98 (d,2.6), 4.07 (t,5.9)
-CH_2-CH_2-COOH (C3)	1.64 (m)	Sucrose	4.2 (d,8.7), 5.4 (d,3.8)
-CH_2-COOH (C2)	2.36 (t,7.5)	Erythrose	4.4 (m)
**Amino acids**		β-glucose	4.64 (d,7.9)
Leucine	0.94 (t,5.9), 1.70 (m)	α-glucose	5.22 (d,3.7)
Valine	0.97 (d,7.0), 1.02 (d,7.1), 2.29 (m)	Maltose	3.90 (m), 5.41 (dd)
		**Organic acids**	
Isoleucine	0.99 (d, 6.9)	Propionic acid	2.16 (q,7.5)
Threonine	1.31 (d,6.5), 3.55 (d,4.8), 4.24 (m)	Lactic acid	1.32 (d,6.9), 4.04 (q,6.9)
Alanine	1.47 (d,7.2)	Acetic acid	1.91 (s)
Arginine	1.68 (m), 1.90 (m)	Succinic acid	2.41 (s)
Lysine	1.71 (m), 1.87 (m)	Citric acid	2.53 (d), 2.65 (d)
Serine	3.84 (dd,5.6,3.8), 3.96 (m)	Fumaric acid	6.51 (s)
		Malic acid	2.39, 2.67 (dd)
Proline	1.99 (m), 2.06 (m), 4.12 (dd,8.6,6.4)	**Others**	
		Choline	3.22 (s)
Glutamic acid	2.04 (m), 2.34 (m)	Creatine	3.92 (s)
Glutamine	2.44 (m)	Myoinositol	4.05 (t,2.8)
Tyrosine	6.87 (m), 7.17 (m)	NAD	6.03 (d,5.8), 6.08 (d,5.7), 6.12 (d,5.8), 8.16 (s), 8.20 (m), 8.44 (s), 8.83 (d,8.0), 9.15 (d,6.3), 9.33 (s)
Histidine	7.09 (d,0.6), 7.90 (d,1.1)	3-hydroxykynurenine	6.7 (t), 7.43 (d)
Tryptophan	7.31 (s), 7.53 (d,8.1), 7.68 (d,8.0)	AMP	8.26 (s), 8.59 (s), 4.81 (t)
Phenylalanine	7.36 (m), 7.41 (m)	ADP	8.54 (s), 4.77 (t)
