# Supplementary material for: Evolution of the metabolome in response to selection for increased immunity in populations of Drosophila melanogaster
Source: PLoS One. 2017 Nov 17;12(11):e0188089. doi: 10.1371/journal.pone.0188089 (PMC5693281; doi:10.1371/journal.pone.0188089)
Supplement: S2 Table — (PDF) [file pone.0188089.s013.pdf]

Metabolite	Peak (in ppm)	Quantity present in					
		S*u*	I*u*	S*i*	I*i*	S*s*	I*s*
Fatty acids	1.27-1.31	3.7257 ± 0.180	5.1829 ± 0.197	2.3213 ± 0.133	3.7090 ± 0.191	2.8794 ± 0.172	4.5624 ± 0.265
Succinate	2.41-2.42	2.3229± 0.091	3.2223 ± 0.178	1.5211 ± 0.067	2.5763 ± 0.067	1.9147 ± 0.090	2.9840 ± 0.113
Citrate	2.53-2.55	0.9230 ± 0.058	1.2655 ± 0.104	0.4287 ± 0.042	0.8523 ± 0.093	0.6090 ± 0.036	1.0678 ± 0.093
Glucose	4.62-4.65	16.7311 ± 0.314	20.9316± 0.226	13.3922 ± 0.204	17.5509± 0.226	14.5910± 0.186	19.3311± 0.136
Galactose	4.07-4.08	2.8101 ± 0.102	3.2251 ± 0.053	2.5642 ± 0.049	2.9806 ± 0.060	2.7772 ± 0.112	3.1641 ± 0.065
Leucine	0.95-0.97	1.2085 ± 0.030	1.3640 ± 0.034	1.4400 ± 0.023	1.5158 ± 0.042	1.3310 ± 0.018	1.4140 ± 0.024
Proline	2.33-2.38	1.9611 ± 0.112	2.5764 ± 0.174	2.5740 ± 0.247	3.0023 ± 0.267	2.1321 ± 0.162	2.7140 ± 0.247
Threonine	4.24-4.28	2.0381 ± 0.110	2.3473 ± 0.069	2.5381 ± 0.149	2.5294 ± 0.165	2.2683 ± 0.149	2.4388 ± 0.149
Lysine	3.00-3.02	0.5996 ± 0.063	0.7824 ± 0.051	0.8457 ± 0.052	0.9466 ± 0.059	0.6857 ± 0.046	0.8466 ± 0.041
Arginine	1.60-1.75	1.6998 ± 0.079	1.8342 ± 0.069	1.9613 ± 0.081	2.0872 ± 0.054	1.7606 ± 0.091	1.9462 ± 0.038
Alanine	1.46-1.50	1.6931± 0.052	1.8152 ± 0.055	2.3646 ± 0.029	1.9674 ± 0.051	1.7931 ± 0.050	1.8852 ± 0.059
Myoinositol	4.05-4.07	5.3274 ± 0.141	5.4746 ± 0.073	5.9279 ± 0.131	6.0163 ± 0.130	5.4474 ± 0.158	5.6846 ± 0.060
Lactate	1.32-1.33	0.5970 ± 0.127	0.6746 ± 0.082	1.2836 ± 0.106	0.9638 ± 0.045	0.6570 ± 0.082	0.7246 ± 0.073
NAD	8.44-8.45	0.3690 ± 0.038	0.4946 ± 0.035	0.6523 ± 0.073	0.7113 ± 0.043	0.4632 ± 0.082	0.5632 ± 0.013
AMP	8.59-8.60	0.2647 ± 0.024	0.3683 ± 0.065	0.4962 ± 0.075	0.6899 ± 0.085	0.3375 ± 0.033	0.4962 ± 0.061
Sterol	0.76-0.77	0.2099 ± 0.023	0.2346 ± 0.037	0.1682 ± 0.028	0.2235 ± 0.021	0.1940 ± 0.042	0.2091 ± 0.022
Valine	0.97-0.98	4.1975 ± 0.189	4.284 ± 0.338	3.8902 ± 0.173	3.9896 ± 0.246	3.9902 ± 0.126	4.0214 ± 0.263
Isoleucine	0.99-1.00	2.3493 ± 0.110	2.1582 ± 0.441	2.5515 ± 0.363	2.4792 ± 0.095	2.4047 ± 0.131	2.3781 ± 0.208
Glutamine	2.44-2.46	2.3864 ± 0.174	2.5011 ± 0.166	2.4122 ± 0.083	2.5891 ± 0.165	2.4801 ± 0.287	2.3415 ± 0.278
Glutamic acid	2.34-2.35	4.5396 ± 0.161	4.6971 ± 0.151	4.6387 ± 0.248	4.5591 ± 0.391	4.3575 ± 0.350	4.4102 ± 0.319
Histidine	7.09-7.11	0.9994 ± 0.069	1.0449 ± 0.115	0.9343 ± 0.031	0.9639 ± 0.197	0.9937 ± 0.099	0.9818 ± 0.052
Tyrosine	7.17-7.18	0.1947 ± 0.012	0.2104 ± 0.016	0.2418 ± 0.023	0.3151 ± 0.041	0.2581 ± 0.024	0.2934 ± 0.023

Tryptophan	7.31-7.32	0.6221 ± 0.031	0.5585 ± 0.032	0.6892 ± 0.041	0.3832 ± 0.026	0.5970 ± 0.020	0.4598 ± 0.033
Phenylalanine	7.36-7.37	0.2741 ± 0.021	0.2106 ± 0.027	0.3674 ± 0.061	0.3346 ± 0.049	0.3314 ± 0.015	0.2699 ± 0.057
3-OHkynurenine	6.70-6.72	0.1983 ± 0.022	0.2029 ± 0.024	0.2342 ± 0.018	0.1902 ± 0.025	0.1961 ± 0.024	0.2001 ± 0.013
ADP	8.54-8.55	0.6498 ± 0.016	0.6573 ± 0.041	0.7060 ± 0.030	0.7172 ± 0.069	0.6590 ± 0.020	0.6963 ± 0.016
Creatine	3.92-3.93	1.2127 ± 0.090	1.2137 ± 0.103	1.3561 ± 0.059	1.5364 ± 0.080	1.2827 ± 0.219	1.4337 ± 0.105
Choline	3.22-3.23	1.1023 ± 0.153	1.1622 ± 0.087	1.5477 ± 0.165	1.4594 ± 0.272	1.3732 ± 0.093	1.3753 ± 0.209
Malic acid	2.67-2.68	0.4739 ± 0.036	0.5777 ± 0.066	0.4640 ± 0.081	0.5179 ± 0.119	0.4217 ± 0.118	0.5170 ± 0.070
Fumaric acid	6.51-6.52	0.1095 ± 0.049	0.0928 ± 0.009	0.1698 ± 0.057	0.1217 ± 0.066	0.1195 ± 0.021	0.1082 ± 0.033
Propionic acid	2.16-2.17	2.4153 ± 0.059	2.7095 ± 0.057	2.8831 ± 0.136	2.9317 ± 0.068	2.7273 ± 0.063	2.8979 ± 0.071
Sucrose	5.40-5.41	3.1938 ± 0.108	4.0896 ± 0.152	3.0215 ± 0.079	3.7045 ± 0.072	3.1546 ± 0.074	3.8058 ± 0.060
