# Supplementary material for: Evolution of the metabolome in response to selection for increased immunity in populations of Drosophila melanogaster
Source: PLoS One. 2017 Nov 17;12(11):e0188089. doi: 10.1371/journal.pone.0188089 (PMC5693281; doi:10.1371/journal.pone.0188089)
Supplement: S3 Table — (PDF) [file pone.0188089.s014.pdf]

Metabolite		Selection	Treatment	Selection X Treatment
Fatty acids	F	18.11	3.07	3.35
	Q	**<0.001**	0.058	0.053
Glucose	F	15.55	5.01	1.54
	Q	**<0.001**	0.053	0.063
Galactose	F	3.53	2.09	0.13
	Q	**0.045**	0.161	0.871
Sucrose	F	12.02	2.45	1.17
	Q	**<0.001**	0.932	0.138
Malate	F	3.93	4.03	0.02
	Q	**0.033**	0.056	0.983
Citrate	F	3.96	4.10	1.02
	Q	0.058	**0.029**	0.373
Succinate	F	8.79	5.63	2.86
	Q	**0.006**	**0.010**	0.077
Proline	F	15.72	7.60	4.49
	Q	**<0.001**	**0.002**	**0.040**
Arginine	F	12.83	12.47	5.32
	Q	**<0.001**	**<0.001**	**0.037**
Leucine	F	7.32	6.38	7.12
	Q	**0.003**	**0.047**	**0.038**
Lysine	F	5.73	2.98	4.93
	Q	**0.029**	**0.049**	**0.037**
Histidine	F	4.19	2.32	0.54
	Q	**0.002**	0.140	0.589
NAD	F	10.26	4.31	5.47
	Q	**0.003**	0.065	0.370
AMP	F	10.78	10.65	1.03
	Q	**0.003**	**<0.001**	0.370
ADP	F	6.15	0.68	2.12
	Q	**0.007**	0.512	0.631
3-hydroxykynurenine	F	4.07	2.62	0.14
	Q	**0.030**	0.118	0.861
Tyrosine	F	5.48	1.47	0.27
	Q	**0.011**	0.247	0.607
Tryptophan	F	3.57	3.38	1.31
	Q	**0.044**	0.078	0.287
Phenylalanine	F	1.89	6.17	0.14

	Q	0.181	**0.007**	0.862
Glutamate	F	6.76	0.53	0.02
	Q	**0.004**	0.595	0.883
Alanine	F	0.25	3.50	2.80
	Q	0.615	**0.046**	0.051
Lactate	F	2.46	4.50	3.44
	Q	0.129	**0.022**	0.058
Threonine	F	1.25	2.71	2.96
	Q	**0.003**	**0.034**	**0.007**
Trehalose	F	0.61	0.91	1.62
	Q	0.440	0.414	0.218
Ribose	F	0.21	0.37	0.04
	Q	0.728	0.694	0.959
Erythrose	F	3.31	2.93	0.14
	Q	0.081	0.074	0.863
Maltose	F	0.22	0.50	0.12
	Q	0.636	0.612	0.884
Propionate	F	1.53	1.97	0.47
	Q	0.228	0.162	0.628
Acetate	F	2.58	0.93	0.10
	Q	0.121	0.496	0.898
Fumarate	F	0.42	0.11	2.86
	Q	0.521	0.896	0.077
Valine	F	0.44	1.53	1.43
	Q	0.510	0.237	0.259
Isoleucine	F	0.41	2.97	1.34
	Q	0.526	0.070	0.280
Serine	F	1.95	1.87	0.28
	Q	0.174	0.176	0.756
Glutamine	F	3.99	2.74	1.28
	Q	0.057	0.085	0.296
Choline	F	0.02	1.04	0.18
	Q	0.884	0.366	0.830
Creatine	F	0.28	0.97	2.29
	Q	0.596	0.393	0.123
Myoinositol	F	0.55	0.71	0.07
	Q	0.462	0.501	0.927
