# Supplementary material for: Evolution of the metabolome in response to selection for increased immunity in populations of Drosophila melanogaster
Source: PLoS One. 2017 Nov 17;12(11):e0188089. doi: 10.1371/journal.pone.0188089 (PMC5693281; doi:10.1371/journal.pone.0188089)
Supplement: S4 Table — (PDF) [file pone.0188089.s015.pdf]

| Metabolite | | Selection | Treatment | Selection X Treatment |
|---|---|---|---|---|
| Fatty acids | F | 7.14 | 5.18 | 0.44 |
| | Q | **0.048** | 0.061 | 0.767 |
| Glucose | F | 13.31 | 0.31 | 1.13 |
| | Q | **<0.001** | 0.732 | 0.339 |
| Galactose | F | 17.71 | 1.03 | 1.24 |
| | Q | **<0.001** | 0.371 | 0.305 |
| Sucrose | F | 13.93 | 0.94 | 1.68 |
| | Q | **<0.001** | 0.403 | 0.206 |
| Malate | F | 4.05 | 4.82 | 0.30 |
| | Q | 0.055 | **0.017** | 0.742 |
| Citrate | F | 9.68 | 11.32 | 0.01 |
| | Q | **<0.001** | **<0.001** | 0.983 |
| Succinate | F | 5.27 | 2.43 | 0.59 |
| | Q | **0.030** | 0.109 | 0.560 |
| Proline | F | 8.14 | 9.44 | 0.88 |
| | Q | **0.008** | **<0.001** | 0.425 |
| Arginine | F | 3.79 | 4.90 | 4.47 |
| | Q | **0.029** | **0.016** | **0.022** |
| Leucine | F | 45.08 | 6.04 | 3.47 |
| | Q | **<0.001** | **0.007** | **0.047** |
| Lysine | F | 0.279 | 4.19 | 4.27 |
| | Q | 0.602 | **0.014** | **0.011** |
| Histidine | F | 3.34 | 0.16 | 0.57 |
| | Q | 0.079 | 0.852 | 0.572 |
| NAD | F | 4.71 | 5.11 | 3.67 |
| | Q | **0.039** | **0.014** | 0.061 |
| AMP | F | 5.68 | 6.00 | 0.16 |
| | Q | **0.009** | **0.007** | 0.688 |
| ADP | F | 10.08 | 4.32 | 2.52 |
| | Q | **0.004** | **0.024** | 0.101 |
| 3-hydroxykynurenine | F | 7.75 | 0.27 | 1.03 |
| | Q | **0.010** | 0.760 | 0.369 |
| Tyrosine | F | 5.88 | 7.93 | 0.28 |
| | Q | **0.023** | **0.002** | 0.754 |
| Tryptophan | F | 3.85 | 3.56 | 0.34 |
| | Q | **0.035** | 0.071 | 0.709 |
| Phenylalanine | F | 20.36 | 8.80 | 0.91 |

| | | | | |
|---|---|---|---|---|
| | Q | **<0.001** | **0.002** | 0.416 |
| Glutamate | F | 5.62 | 0.079 | 1.81 |
| | Q | **0.009** | 0.781 | 0.184 |
| Alanine | F | 1.84 | 8.02 | 4.61 |
| | Q | 0.179 | **0.002** | 0.052 |
| Lactate | F | 4.06 | 7.35 | 0.65 |
| | Q | 0.055 | **0.003** | 0.527 |
| Threonine | F | 19.42 | 3.61 | 14.52 |
| | Q | **<0.001** | **0.042** | **<0.001** |
| Trehalose | F | 0.02 | 0.48 | 2.52 |
| | Q | 0.872 | 0.619 | 0.101 |
| Ribose | F | 0.04 | 0.57 | 1.53 |
| | Q | 0.829 | 0.572 | 0.235 |
| Erythrose | F | 1.05 | 2.05 | 0.41 |
| | Q | 0.315 | 0.150 | 0.663 |
| Maltose | F | 1.72 | 0.92 | 0.51 |
| | Q | 0.201 | 0.412 | 0.601 |
| Propionate | F | 1.80 | 2.37 | 0.34 |
| | Q | 0.191 | 0.114 | 0.715 |
| Acetate | F | 0.15 | 0.34 | 0.21 |
| | Q | 0.694 | 0.710 | 0.807 |
| Fumarate | F | 0.46 | 0.83 | 1.10 |
| | Q | 0.503 | 0.445 | 0.348 |
| Valine | F | 1.05 | 1.14 | 1.01 |
| | Q | 0.314 | 0.335 | 0.380 |
| Isoleucine | F | 3.09 | 0.33 | 1.81 |
| | Q | 0.091 | 0.716 | 0.184 |
| Serine | F | 0.36 | 0.07 | 1.93 |
| | Q | 0.549 | 0.926 | 0.166 |
| Glutamine | F | 0.02 | 1.47 | 1.79 |
| | Q | 0.965 | 0.249 | 0.187 |
| Choline | F | 0.48 | 1.36 | 1.18 |
| | Q | 0.614 | 0.336 | 0.846 |
| Creatine | F | 1.29 | 1.50 | 0.64 |
| | Q | 0.265 | 0.242 | 0.535 |
| Myoinositol | F | 0.48 | 0.73 | 0.95 |
| | Q | 0.510 | 0.492 | 0.398 |
