# Supplementary material for: Evolution of the metabolome in response to selection for increased immunity in populations of Drosophila melanogaster
Source: PLoS One. 2017 Nov 17;12(11):e0188089. doi: 10.1371/journal.pone.0188089 (PMC5693281; doi:10.1371/journal.pone.0188089)
Supplement: S5 Table — (PDF) [file pone.0188089.s016.pdf]

Metabolite		Selection	Treatment	Selection X Treatment
Fatty acids	F	16.73	2.70	0.48
	Q	**<0.001**	0.087	0.619
Glucose	F	6.94	1.15	0.59
	Q	**0.014**	0.333	0.559
Galactose	F	4.40	0.83	0.25
	Q	**0.046**	0.445	0.778
Sucrose	F	6.57	0.33	2.65
	Q	**0.017**	0.722	0.090
Malate	F	0.07	3.03	0.76
	Q	0.790	0.067	0.475
Citrate	F	2.10	5.18	0.79
	Q	0.159	**0.013**	0.464
Succinate	F	0.10	3.84	0.71
	Q	0.750	**0.035**	0.497
Proline	F	4.34	2.61	0.63
	Q	**0.047**	0.094	0.538
Arginine	F	5.53	4.20	4.75
	Q	**0.027**	**0.048**	**0.031**
Leucine	F	12.69	3.47	0.61
	Q	**<0.001**	**0.047**	0.547
Lysine	F	6.07	3.34	4.27
	Q	**<0.001**	**0.045**	**0.031**
Histidine	F	5.03	2.93	0.99
	Q	**0.034**	0.072	0.384
NAD	F	14.64	3.92	0.21
	Q	**<0.001**	**0.033**	0.805
AMP	F	7.39	5.35	3.08
	Q	**0.011**	**0.012**	0.064
ADP	F	6.31	3.90	1.08
	Q	**0.019**	**0.033**	0.355
3-hydroxykynurenine	F	6.97	3.34	0.02
	Q	**0.014**	0.052	0.975
Tyrosine	F	9.17	0.44	0.25
	Q	**0.005**	0.644	0.774
Tryptophan	F	13.77	0.47	0.25
	Q	**0.001**	0.629	0.774
Phenylalanine	F	15.37	0.19	2.57

	Q	**<0.001**	0.821	0.096
Glutamate	F	4.94	0.12	2.48
	Q	**0.015**	0.727	0.104
Alanine	F	0.48	12.57	3.64
	Q	0.622	**0.001**	**0.041**
Lactate	F	0.57	5.43	1.05
	Q	0.454	**0.011**	0.362
Threonine	F	20.04	3.52	6.73
	Q	**<0.001**	**0.045**	**0.004**
Trehalose	F	1.83	1.37	0.24
	Q	0.188	0.272	0.783
Ribose	F	1.92	0.14	0.04
	Q	0.177	0.865	0.959
Erythrose	F	1.04	2.48	3.23
	Q	0.317	0.104	0.056
Maltose	F	2.55	0.06	1.37
	Q	0.123	0.937	0.271
Propionate	F	3.41	2.20	0.08
	Q	0.077	0.131	0.918
Acetate	F	0.21	0.71	2.72
	Q	0.652	0.498	0.085
Fumarate	F	0.08	2.48	2.23
	Q	0.768	0.104	0.129
Valine	F	0.02	1.01	0.48
	Q	0.872	0.378	0.620
Isoleucine	F	0.05	2.63	0.77
	Q	0.826	0.092	0.473
Serine	F	0.33	2.24	1.49
	Q	0.569	0.128	0.244
Glutamine	F	0.68	1.26	1.52
	Q	0.416	0.301	0.237
Choline	F	0.27	3.84	1.41
	Q	0.607	**0.035**	0.26
Creatine	F	0.96	1.79	1.56
	Q	0.337	0.188	0.229
Myoinositol	F	1.14	3.76	1.80
	Q	0.294	**0.037**	0.185
