# Supplementary material for: Evolution of the metabolome in response to selection for increased immunity in populations of Drosophila melanogaster
Source: PLoS One. 2017 Nov 17;12(11):e0188089. doi: 10.1371/journal.pone.0188089 (PMC5693281; doi:10.1371/journal.pone.0188089)
Supplement: S6 Table — (PDF) [file pone.0188089.s017.pdf]

Metabolite		Selection	Treatment	Selection X Treatment
Fatty acids	F	7.12	0.51	1.99
	Q	**0.003**	0.606	0.170
Glucose	F	7.41	2.37	1.34
	Q	**0.011**	0.114	0.279
Galactose	F	4.51	1.36	0.56
	Q	**0.044**	0.273	0.574
Sucrose	F	5.03	1.35	0.27
	Q	**0.034**	0.278	0.762
Malate	F	5.06	2.72	1.68
	Q	**0.033**	0.085	0.206
Citrate	F	8.20	18.02	3.92
	Q	0.051	**<0.001**	0.054
Succinate	F	4.41	3.60	2.18
	Q	**0.046**	**0.042**	0.134
Proline	F	9.82	5.71	4.68
	Q	**0.004**	**0.009**	**0.019**
Arginine	F	7.31	8.34	5.01
	Q	**0.005**	**0.001**	**0.016**
Leucine	F	4.92	5.38	7.18
	Q	**0.036**	**0.027**	**0.032**
Lysine	F	4.83	5.98	4.33
	Q	**0.031**	**0.029**	**0.037**
Histidine	F	4.43	0.33	3.21
	Q	**0.045**	0.720	0.058
NAD	F	6.45	0.05	0.95
	Q	**0.017**	0.944	0.398
AMP	F	15.74	11.06	2.09
	Q	**<0.001**	**<0.001**	0.144
ADP	F	8.34	2.43	1.01
	Q	**0.001**	0.131	0.376
3-hydroxykynurenine	F	3.41	0.41	1.76
	Q	**0.049**	0.867	0.196
Tyrosine	F	4.41	3.10	1.53
	Q	**0.023**	0.062	0.227
Tryptophan	F	3.99	0.01	1.20
	Q	**0.031**	0.908	0.316
Phenylalanine	F	0.02	5.01	1.41

	Q	0.868	**0.015**	0.262
Glutamate	F	7.68	0.72	0.68
	Q	**0.002**	0.499	0.989
Alanine	F	0.02	5.37	0.69
	Q	0.866	**0.011**	0.507
Lactate	F	0.02	7.36	1.92
	Q	0.887	**0.003**	0.167
Threonine	F	1.05	2.68	3.31
	Q	**0.023**	**0.014**	**0.007**
Trehalose	F	0.41	2.34	0.43
	Q	0.525	0.117	0.655
Ribose	F	0.05	1.03	0.22
	Q	0.901	0.371	0.803
Erythrose	F	0.66	1.05	1.68
	Q	0.422	0.363	0.206
Maltose	F	0.01	0.10	1.16
	Q	0.919	0.903	0.329
Propionate	F	0.01	0.64	0.65
	Q	0.991	0.533	0.527
Acetate	F	0.08	0.43	0.30
	Q	0.773	0.653	0.743
Fumarate	F	1.40	1.14	0.54
	Q	0.248	0.334	0.584
Valine	F	0.52	0.82	1.04
	Q	0.473	0.448	0.367
Isoleucine	F	0.98	1.19	0.44
	Q	0.330	0.320	0.647
Serine	F	0.04	0.30	0.65
	Q	0.946	0.738	0.530
Glutamine	F	0.60	2.70	0.05
	Q	0.445	0.087	0.950
Choline	F	1.40	1.11	1.28
	Q	0.247	0.344	0.294
Creatine	F	0.04	0.92	0.21
	Q	0.825	0.410	0.813
Myoinositol	F	0.10	1.37	0.37
	Q	0.746	0.272	0.691
